# Past and future potential range changes in one of the last large vertebrates of the Australian continent, the emu *Dromaius novaehollandiae*

**DOI:** 10.1038/s41598-020-79551-0

**Published:** 2021-01-13

**Authors:** Julia Ryeland, Tristan T. Derham, Ricky J. Spencer

**Affiliations:** 1 Hawkesbury Institute for the Environment, Western Sydney University, Hawkesbury Campus, Locked Bag 1797, Penrith, NSW 2751 Australia; 2 School of Natural Sciences, University of Tasmania, Private Bag 55, Hobart, 7001 Australia

**Keywords:** Biogeography, Ecological modelling, Climate-change ecology

## Abstract

In Australia, significant shifts in species distribution have occurred with the loss of megafauna, changes in indigenous Australian fire regime and land-use changes with European settlement. The emu, one of the last megafaunal species in Australia, has likely undergone substantial distribution changes, particularly near the east coast of Australia where urbanisation is extensive and some populations have declined. We modelled emu distribution across the continental mainland and across the Great Dividing Range region (GDR) of eastern Australia, under historical, present and future climates. We predicted shifts in emu distribution using ensemble modelling, hindcasting and forecasting distribution from current emu occurrence data. Emus have expanded their range northward into central Australia over the 6000 years modelled here. Areas west of the GDR have become more suitable since the mid-Holocene, which was unsuitable then due to high precipitation seasonality. However, the east coast of Australia has become climatically sub-optimal and will remain so for at least 50 years. The north east of NSW encompasses the range of the only listed endangered population, which now occurs at the margins of optimal climatic conditions for emus. Being at the fringe of suitable climatic conditions may put this population at higher risk of further decline from non-climatic anthropogenic disturbances e.g. depredation by introduced foxes and pigs. The limited scientific knowledge about wild emu ecology and biology currently available limits our ability to quantify these risks.

## Introduction

The current distribution and habitat associations of species are generally determined by past changes in climate and environment^1,2^. Current distribution and habitat association in turn drive changes in future distribution^3,4^. Despite this, past distributions are often left unexplored when modelling habitat suitability and species range^5^. In Australia, the landscape changed dramatically with the arrival of Aboriginal people (ca. 65 kyr^6,7^), the loss of most megafauna (by ca. 40–45 kya^8–11^), and with European settlement (from 1787^12^). Many species have experienced range shifts in response to altered fire regimes and hunting since indigenous Australian arrival, changes in the structure of habitat with megafauna extinction (with woody vegetation no longer strongly suppressed^8,10,11^) and with anthropogenic influence since European settlement (e.g. increasing urbanisation, the introduction of predators and competitors, and land clearing^13,14^). In particular, the geographic range and foraging strategies of Australia’s extant large herbivores have likely changed with the loss of competition from other herbivorous megafauna (i.e. species > 44 kg)^10,11^. The interpretation of long-term shifts in a species’ range, in response to ecological changes, can inform management of such species under climate change scenarios.

The present effects of historical shifts in species distribution across Australia are most evident for species from ancient lineages such as the *Casuariidae, Macropodidae* and *Vombatidae*^11^. The emu, *Dromaius novaehollandiae*, and cassowary species, *Casuarius spp*., are the last of the *Casuariidae*, -  a family of flightless, large-bodied and predominately herbivorous birds^15–17^. The emu has a generalist omnivorous diet (mainly consuming plants^18,19^), and is known to be an important non-standard dispersal agent (i.e. agents to which the plant species is not apparently adapted^20^), transporting and germinating seeds from plants with a wide range of dispersal syndromes over large distances^21,22^. The dispersal of seeds by emus is thought to influence plant population structure and gene flow, plant colonization opportunities, range expansion rates, and speciation and extinction rates^16,23^; as is the case with other large-bodied seed dispersers^24–27^. Being one of the last remaining Australian megafaunal species and occurring across most of the continent (unlike the cassowary which is restricted to the northern wet tropics), the emu likely plays an important role in ecosystem connectivity and structure; a role which would be unfilled if the emu disappeared from Australian landscapes. However, relatively little is known about emu movement, population structure or historical changes in distribution.

Emu distribution has shifted with climatic changes and anthropogenic effects on the landscape. The extinction of the megafauna likely altered Australian landscapes, in particular, causing expansion of scrubland steppes around 45 kyr^28^. This caused significant shifts in emu diet from a predominately C4 plant-based diet prior to megafauna extinction, to an almost solely C3 plant-based diet^28^, potentially driving emus into areas with higher availability of C3 plants. Anthropogenic change since European colonisation has also likely altered their distribution, with an increase in both agricultural land uses and urban areas. Emus were intensely hunted by early Europeans and persecuted as an agricultural pest through to the twentieth century^29^, decreasing their numbers and even driving them to local extinction, such as across the island state of Tasmania^30,31^. There have been no previous attempts to quantify changes in emu distribution across Australia. However, many anecdotal reports suggest that the distribution and frequency of emus across some regions of Australia has rapidly changed since European settlement, particularly along the Great Diving Range and the areas east of that mountain range (‘GDR’)^32^. In the early twentieth century, emus were reported in large numbers from northern coastal New South Wales (NSW) to coastal Victoria, but emus are now relatively uncommon in those areas^23^. Only one endemic population is known to remain in NSW east of the mountain range—a population listed as endangered under state legislation^32^. Despite the charismatic nature of the bird, important in both European and indigenous histories (a totem to many indigenous groups and present on the Australian coat of arms), little is known about emu behaviour, biology or ecology in the wild. The threats to the emu populations remain unclear and it is not known whether environmental and climatic changes played a role in their disappearance, nor whether emus are likely to continue to decline.

Here, we aimed to model the influence of environmental and climatic factors on emu distribution and to describe distributional changes across Australia, particularly for areas where emus are likely to have declined since colonisation (i.e. east of the GDR). We used a consensus method of species distribution modelling. This method simultaneously applies several methods to create a best fit ‘ensemble model’ of current emu distribution, here performed over a continental domain (Australian mainland) and across the GDR. An important aim was to understand the main drivers of emu distribution, including both climatic and anthropogenic environmental factors as predictors of emu occurrence. We subsequently hindcast (to past mid-Holocene climates, ~ 6000 yra) and forecast (to future climates, year 2070) those predictors to estimate past and future emu distribution. From these predictions, we estimated range changes between the mid-Holocene and today and over the next 50 years to inform emu management. As emus are believed to be declining in the east of the GDR, this will help in identifying populations at risk, habitats for reintroductions and conservation priorities under climate change^33^.

## Results

### Model performance

Algorithms estimating current emu distribution had variable performance when both bioclimatic and other environmental variables were included (Table 1, Supplementary Table S2). However, this was improved by ensemble modelling, after removing models with < 0.7 TSS, < 0.9 AUC or < 0.7 KAPPA (Table 1). Models that included all variables, did not improve model performance over models that included only bioclimatic variables (Table 1). The high performance of climate-only ensemble models was also reflected in the Boyce Index for the Australian mainland model (all variables: 0.94; climate variables: 0.96) and the GDR model (all variables: 0.98, climate variables: 0.99). Whilst there was more variability in the individual models predicting the GDR distribution and therefore fewer individual algorithms used for ensembles (36 and 39 for the Australian mainland and 32 and 22 for the GDR, all variables and climatic-only models respectively), ensemble models performed similarly for both geographic domains (Table 1). As there was no improvement gained by including all variables, climate-only models were used to predict past and future potential distributions of emus in both geographic domains. Sensitivity and specificity of these climate-only ensemble models was high at 88.34% and 92.60% for Australian mainland model and 92.99% and 92.09% for the GDR model, respectively. For calculation and graphic representation of range change, these models produced a binary threshold of 0.61 and 0.40 (based on the value that maximised the TSS), Australian mainland and GDR models, respectively. Here we map predictions of potential past, current and future distributions for the Australian mainland and for the GDR, using climate-only models.

**Table 1:** Model performance test scores (mean ± SD) for each domain, including all variables (‘all’) or including only bioclimatic variables (‘climate-only’).

Geographic domain	Predictors	TSS	AUC	KAPPA	n
**All algorithms**					
Australian mainland	All	0.62 ± 0.26	0.85 ± 0.15	0.62 ± 0.26	54
Australian mainland	Climate-only	0.69 ± 0.10	0.90 ± 0.07	0.70 ± 0.10	54
GDR	All	0.64 ± 0.171	0.86 ± 0.13	0.66 ± 0.16	54
GDR	Climate-only	0.62 ± 0.18	0.85 ± 0.13	0.64 ± 0.18	54
**Algorithms included in ensemble models**					
Australian mainland	All	0.77 ± 0.04	0.94 ± 0.02	0.77 ± 0.04	36
Australian mainland	Climate-only	0.75 ± 0.04	0.93 ± 0.02	0.75 ± 0.04	39
GDR	All	0.74 ± 0.04	0.94 ± 0.02	0.74 ± 0.04	32
GDR	Climate-only	0.75 ± 0.04	0.94 ± 0.02	0.75 ± 0.04	22

Included are the scores for models with all algorithms and ensemble models (including algorithms above the threshold). n is the product of the number of algorithms and the number of model runs per algorithm.

### Drivers of emu distribution

**Figure 1:**
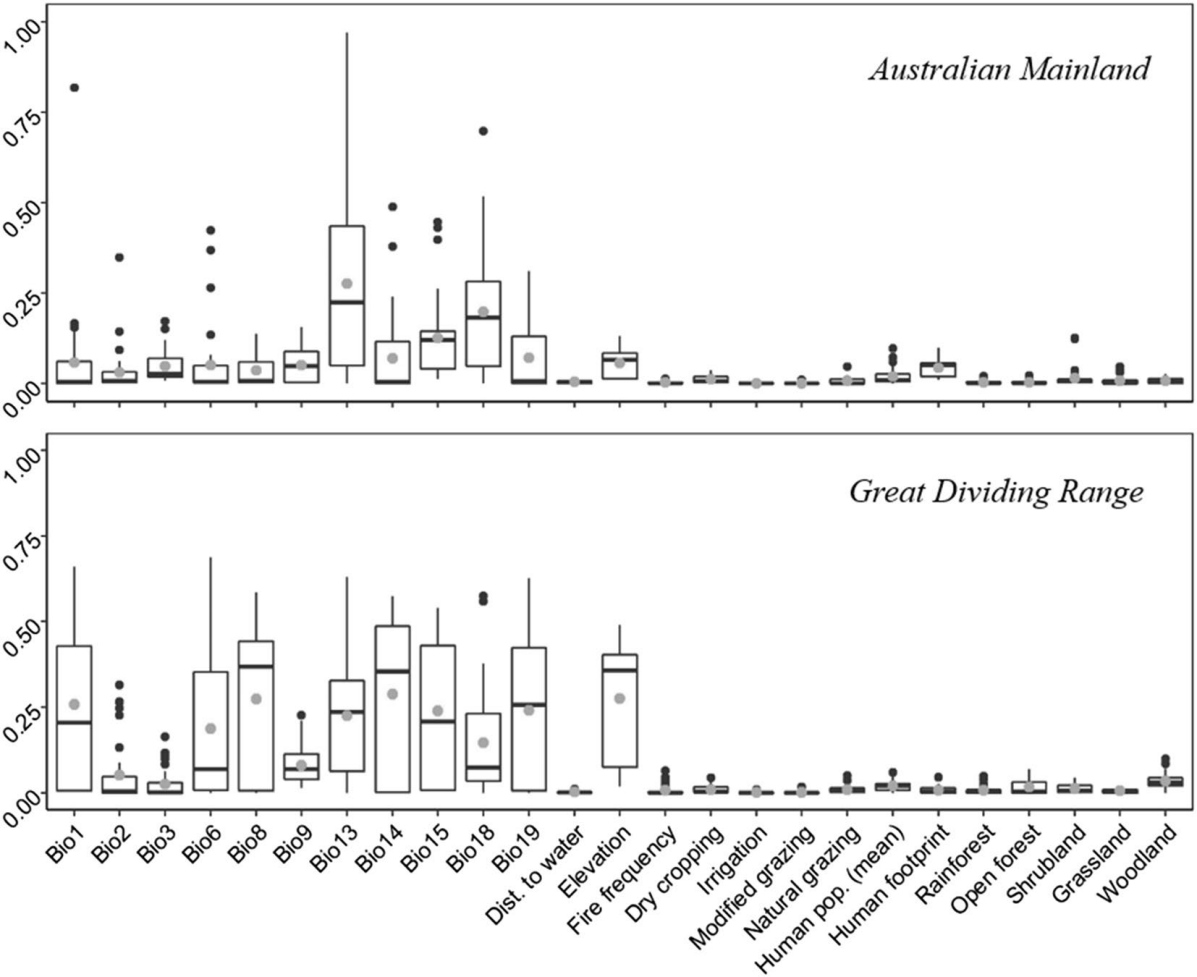
Variable importance for individual algorithms, correlations between projected models and a model with randomized variables, as described in the main text. Higher values indicate predictors that are more important for an algorithm’s predictive performance. Only models with high performance were included (i.e. TSS > 0.7, AUC > 0.98 and Kappa > 0.7). The mean variable importance across models are shown with a grey dot and outliers with black dots. The figures were generated in R (version 3.6.0, https://www.r-project.org/).

**Figure 2:**
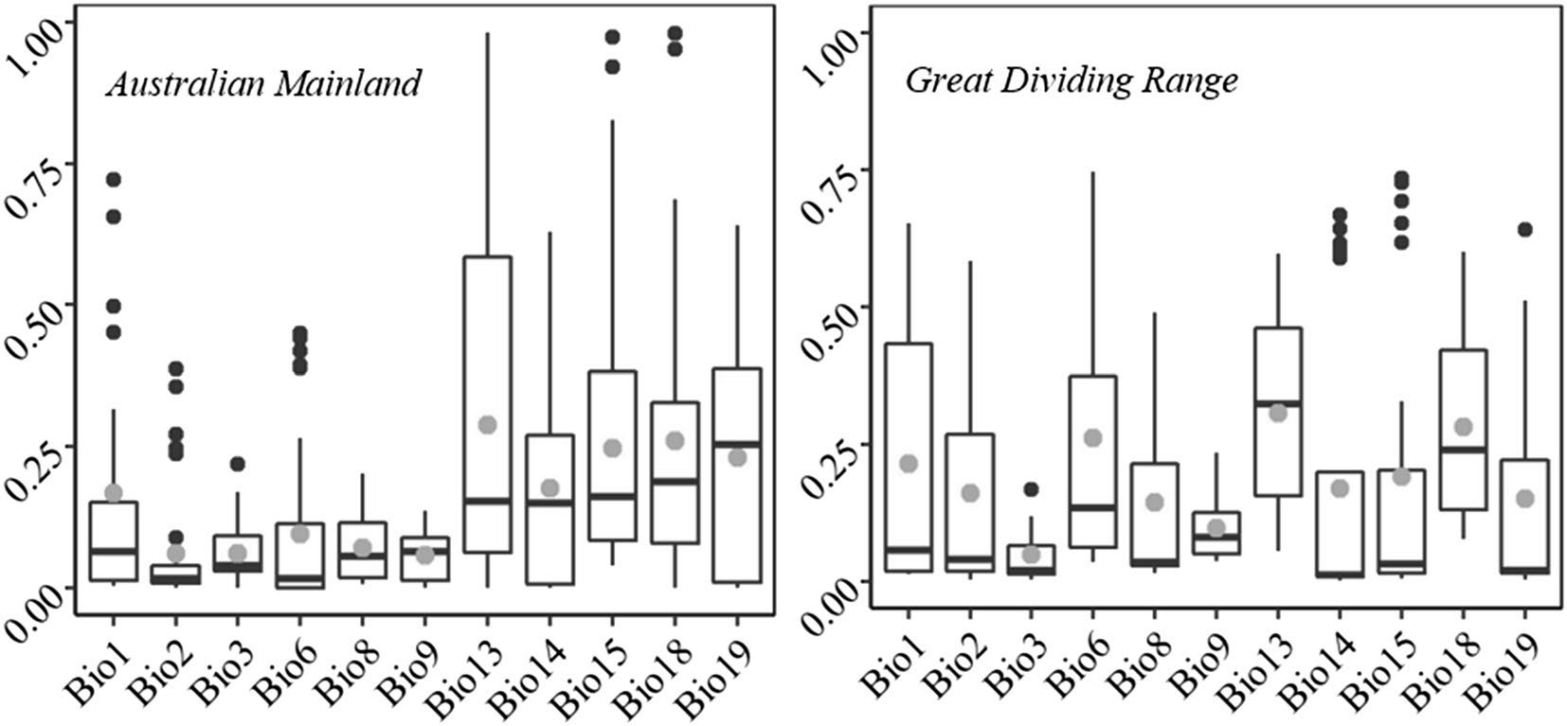
Variable importance for bioclimatic variable only individual algorithms. For description, see Fig. 1. The figures were generated in R (version 3.6.0, https://www.r-project.org/).

While there was variation among models in the assignment of variable importance for current potential distribution, most placed bioclimatic variables as highly important drivers of emu distribution for both the current Australian mainland and GDR range. Over both geographic extents, precipitation patterns predominately drove distribution, with emus being more likely to occur in areas with low precipitation seasonality (bio15), and lower precipitation in the warmest quarter (bio18) (Fig. 1). In the GDR model, precipitation in the wettest month (bio13) was of the highest importance, more so in comparison to the Australian mainland model, with emus completely absent in the north east of the GDR; an area with very high rainfall in summer (i.e. monsoonal precipitation patterns). For the GDR model, elevation also had relatively high importance, which was not the case for the Australian mainland model. As no other non-climatic variables were found to be good predictors of emu potential distribution, we modelled current distribution again, this time removing all non-bioclimatic variables. In these climate-only models, precipitation patterns remained important for predicting distribution (Fig. 2). As model performance was not affected by the removal non-bioclimatic predictors (Table 1), we used bioclimatic variables only for all extrapolations to the past and future. Estimates of bioclimatic variables are also considered more reliable than other variables for extrapolating to past and future scenarios.

### Past, present and future distributions of emus

**Figure 3:**
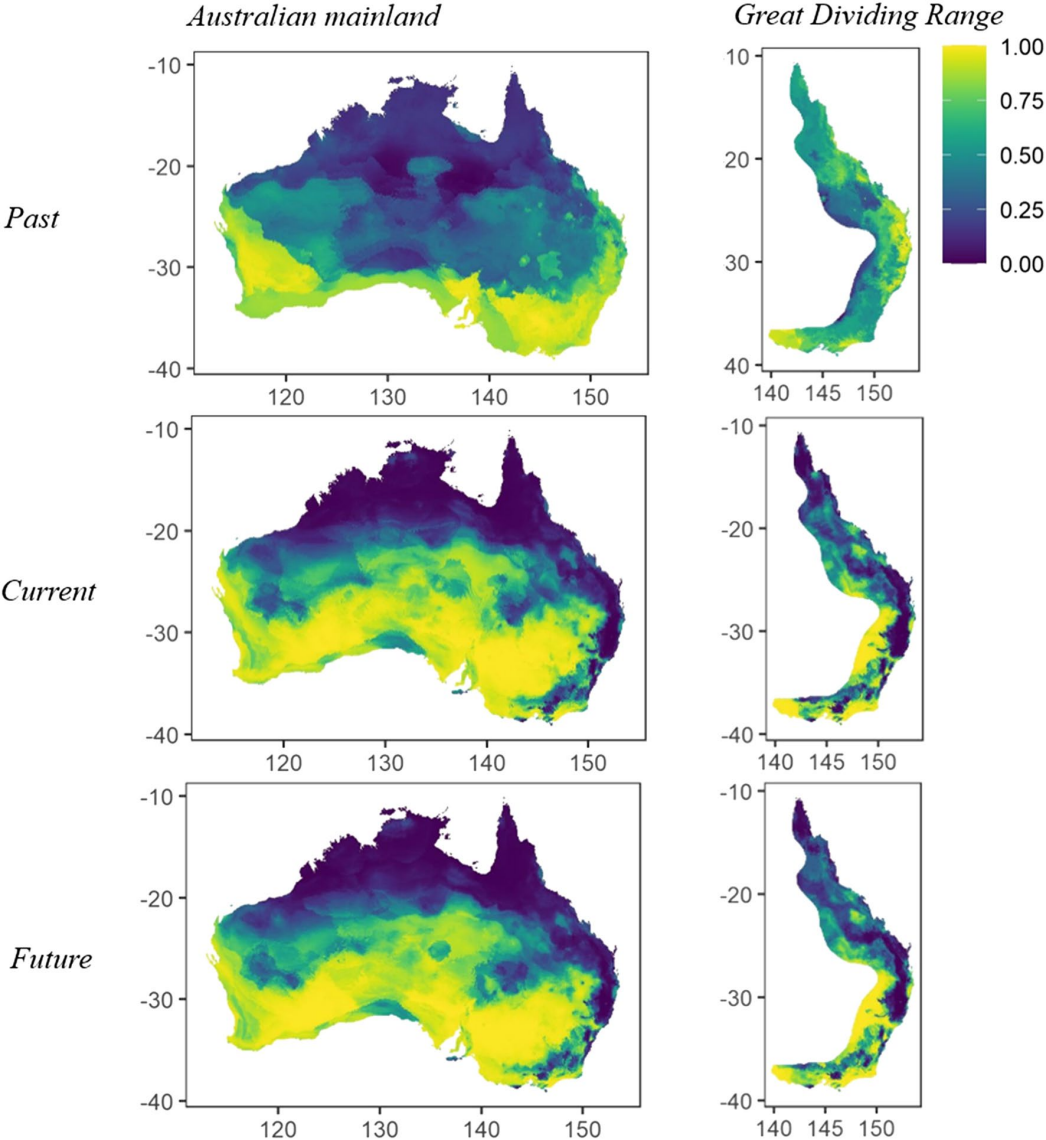
Predicted probability of emu occurrence from ensemble models across the Australian mainland and the Great Dividing Range region, past (~ 6000 yra), present (1970–2020 CE) and future (2070 CE), bioclimatic variables only. Coefficients of variation for each model are presented in Supplementary Fig. S3. The figures were generated in R (version 3.6.0, https://www.r-project.org/).

**Figure 4:**
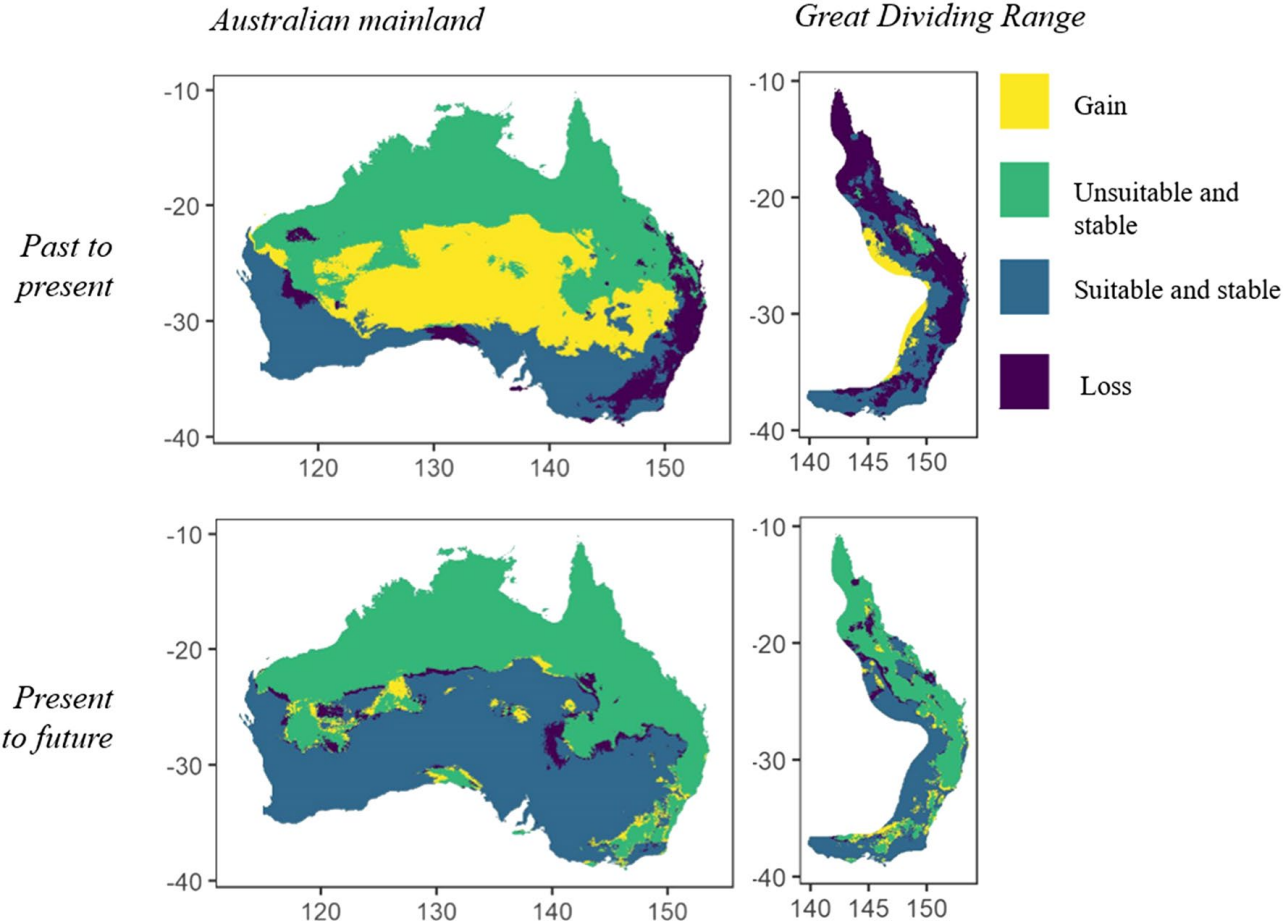
Predicted distribution change from ensemble models, across Australia and the Great Dividing Range region, past to present and present to future. The figures were generated in R (version 3.6.0, https://www.r-project.org/).

Models predicted that large parts of southern Australia had a high likelihood of emu occurrence under the past, current and future climate scenarios. Much of the arid and tropical north of Australia was unsuitable for emus in all models, past, present and future (Fig. 3), though emus are occasionally sighted in these areas. However, whilst similar patterns of occurrence were found in all scenarios, a large northward expansion of emu range across central Australia was predicted to have occurred in response to climatic changes from past and current climate scenarios; though the set of mid-Holocene models had high variance (Supplementary Fig. S3). Although emus are highly mobile and likely to have dispersed under past and future climate change, we present models that do and do not account for dispersal (i.e. movement into newly suitable climates), to provide an estimate of the upper and lower limits of range changes. Whilst climate change may have resulted in a range retraction if dispersal was not possible (an overall decrease in range of 23.05%), with dispersal accounted for, emus were more likely to have increased in range by 80.90% since the mid-Holocene (Fig. 4), particularly into central Australia. However, for the GDR region, models predicted a significant loss in range between the mid-Holocene and the present, with range contractions of between 45.42% and 55.03%, with and without dispersal considered, respectively. The most significant range contraction was mostly observed east of the GDR. Climate change over the next 50 years is unlikely to result in substantial alteration of the current climatically suitable areas across the mainland and GDR, under either the CCSM4 or ACCESS climate change scenarios (Fig. 3, Supplementary Fig. S1, respectively). Future possible emu range across the mainland is predicted to be reduced by between 0.740 and 6.14%, with and without dispersal considered, respectively. Similarly, the range across the GDR will likely remain similar, with a loss of 0.14% and 12.09% predicted, with and without dispersal considered, respectively. These values were derived from the CCSM4 models but similar trends were found when using ACCESS models of future climate potential distribution (Supplementary Fig. S2).

Multivariate environmental similarity surface (MESS) analysis showed that for several predictor climate variables, the mid-Holocene represents conditions outside the range used to analyse current emu distribution, i.e. large areas of the hindcast emu distribution extrapolate beyond current conditions (Supplementary Figure S3). However, climate variables with the highest contribution to current distribution models (bio1, 13, 18 and 19), also showed the least extrapolation in past climate scenarios, for which an interpretation can be given with more certainty (Supplementary Figure S4). Unique combinations of climate patterns may also have occurred in the mid-Holocene, with correlations between climate variables existing within the past climate scenarios that do not occur today or in future scenarios (for example, isothermality is negatively correlated in past scenarios but is positively correlated in current and future climates; Supplementary Figure S6). As such, predictions of past distribution and distribution changes should be viewed with some caution.

## Discussion

Generalist species, particularly large bodied omnivores, often have large distributions extending across a broad range of habitat niches^34–36^ and are likely less affected than other species by habitat changes, such as fragmentation and disturbance^37^. This is the case for the emu, with likely distributions being minimally affected by land use and land cover factors (e.g. vegetation type, water bodies, human land use or urbanization) and are likely to benefit from spatially or temporally heterogeneous environments^38–40^. The probability of occurrence across the mainland and the GDR was strongly driven by bioclimatic variables which is common for distribution predictions at continental scales^41,42^, as well as being common for highly mobile generalists, such as the emu^43^.

### Climatic drivers of emu distribution

A correlation between emu occurrence and both precipitation in the wettest month (bio13) and precipitation in the coldest quarter (bio19; winter across much of Australia) may be explained partly through breeding success and chick survival. Rainfall has previously been noted to influence emu reproduction, with egg production thought to increase in response to rainfall prior to incubation^44^. Chicks are precocial and emerge in spring after a 2-month incubation period^45^. It is postulated that rain just prior to or during incubation increases food availability for males commencing incubation and for emerging chicks, subsequently increasing juvenile survival^44^. The driest quarter across much of the emus range coincides with incubation commencement (April^46,47^), but with chicks emerging in the wettest quarter. Areas with rainfall spread relatively evenly across the year (i.e. low precipitation seasonality) may see higher reproductive success and therefore a greater probability of emu occurrence bringing food availability across their breeding season. High rainfall in the warmest quarter (i.e. high summer rains) but low winter rainfall, may bring high food availability for emus at suboptimal times for chick feeding, reducing feed during chick emergence. Higher egg production in areas where rainfall is present in early winter and higher chick survival in areas with rainfall present in spring would explain the correlation between low seasonality and emu occurrence. Areas with high precipitation in the warmest quarter are most suitable for the related southern cassowary (*Casuarius casuarius*)^48,49^ and exclusion by the southern cassowary in these areas may also drive lower emu occurrence. These mechanistic interpretations of correlative relationships should, however, be treated with caution^50^.

Our models support previous findings of increases in emu occurrence with greater environmental heterogeneity^44^ and the ability of large-bodied generalists to persist across a wide range of modified and non-modified environments^37^. This finding does not, however, exclude the possibility of population level differences, with previous research finding that particular populations have specific habitat preferences^19,44,51^. For example, in Western Australia, emu occurrence and density are positively correlated with pastoral land use and are negatively correlated with arid rangelands^51^, but in southern Victoria they more common in open forest and woodland habitats^19,23,52^. Although the low probability of occurrence in arid range lands may be reflected in our findings by the importance of precipitation to emu occurrence, we did not find that farming practices or vegetation types were useful predictors of emu occurrence at the continental scale or across the vast GDR. At the scale and resolution of our analyses, local preference or adaptation to given resource-rich areas, such as preference for areas with high numbers of flowering and fruiting palatable plants, may not be detectable^15,19,22^. Land use and land cover signals may be also diluted by temporal shifts in preference (i.e. across seasons and years), as emus are known to focus on seasonally abundant food items^19,23,53^ and make seasonal movements towards more favourable climatic conditions^43,48,53,54^. Therefore, while bioclimatic factors are important for understanding emu potential distribution, the management of individual populations requires knowledge of the preferences of the specific populations, which should be modelled at local scales^55–57^.

### Distribution stability under climate change

As emu occurrence was strongly driven by climatic conditions, predominately precipitation patterns, predicting past and future range changes in response to climate change may help to identify populations that are at a higher risk of future decline. Highly mobile generalists are often at lower risk of decline under large climatic changes^58,59^, as they can more readily exploit a wide range of habitats and can move more easily across patchy or fragmented landscapes to areas of better suitability^37,58,59^. Extensive climatic changes, such as that observed between the mid-Holocene and present, were predicted to have significantly altered emu occurrence, with range expansion across central Australia but retraction across the coastal regions of New South Wales and southern Queensland. Large expanses of the southern GDR and the central interior during the mid-Holocene had unsuitable precipitation patterns for emus, with high annual rainfall variability and high precipitation in the warmest quarter^60–62^. Whilst these climatic conditions have since retracted to northeastern Queensland, and much of the western GDR now has a high likelihood of occurrence for emus, the eastern GDR is at the margin of climatic suitability for emus. This includes the areas inclusive of the coastal endangered population range. Indeed this area is likely to have once been highly suitable for emus but may no longer be as suitable due to the increases in winter temperatures. Emus have dark green, ground incubated eggs and high ambient temperature is associated with higher embryonic death^63–65^. Additionally, their dispersal into new climatically favourable environments may have been hindered by their low likelihood to occur in areas of high elevation, thereby potentially climatically isolating the endangered population.

However, the causal factors of shifts from high to low suitability of northern coastal NSW may also be due to factors we were unable to model here. Untested ecological changes such as, historic interactions with other species, phenotypic plasticity and adaptation in response to newly available habitats or environments, or barriers to dispersal, require further investigation to determine their influence on emu distributional change, particularly in the northern coastal NSW. Moreover, extrapolations from present to mid-Holocene should be treated with caution, given the difference in past and current temperature patterns^66^. While climatic changes since the mid-Holocene have likely reduced emu occurrence in the GDR, anthropogenic climate change over the next 50 years is unlikely to have a significant impact on the possible distribution of emus on the Australian mainland generally and in the GDR specifically. This is not surprising considering that the emu is already known to exist across a wide range of climatic conditions in Australia, and therefore given our current knowledge of emu ecology and biology, is unlikely to be as risk of local extinction under climate change scenarios. We do not know, however, how emus will cope physiologically with increasing temperatures. At temperatures above 40 °C, emu body temperature and metabolic rates begin to rise^67,68^. Emus can withstand this for short periods, but how well they can withstand and survive through prolonged periods over 40 °C, likely in the future in the arid north, requires further consideration.

### Implications for management

Although our models predict that emus are unlikely to undergo severe range retractions due to future climate change, they may be more susceptible to anthropogenic impacts in areas where they have undergone previous range retractions or where climate suitability is lower, e.g. the margins of their potential distribution. Whilst our models do not indicate a risk of local extinction due to anthropogenic climatic change, any loss of emu populations may have significant impact on the function of the local ecosystems, in particular seed dispersal and propagation of isolated plant communities. These populations may also represent evolutionary significant units (providing unique genes important for species adaptability and conservation)^69,70^, particularly given the separation of east coast populations from other mainland populations by the high elevation of the GDR and climatic isolation. The emu is also culturally important to local human communities. Our limited knowledge about wild emu ecology and biology limits our ability to assess the risk of further declines due to anthropogenic disturbance, such as predation on the eggs and young by introduced predators. We also have limited knowledge of past and current biotic interactions, with no studies currently available on the relationship of emus to competitors. For example, to understand shifts in emu numbers across Australia through deep ecological time, more research is needed on population changes with shifting predator–prey dynamics, from the loss of apex predators such as *Thylacoleo carnifex*, to the arrival of the dingo (*Canis lupus dingo*) and, much later, sheep, cattle, cats and foxes^71^. No studies have examined these issues, and without such knowledge, management of particular populations is difficult. Further studies should focus on these populations at the fringe of suitable emu climatic conditions, such as the endangered coastal population.

Our results demonstrate that emu distribution across the southern parts of the mainland of Australia has likely remained relatively stable from the mid-Holocene until today, with expansion into the interior and retraction from areas east of the GDR. It is likely that currently, climatically suitable habitat will remain available for at least the next 50 years. Potential emu distribution predictions were driven by rainfall patterns, with rainfall across both the incubation period and chick rearing increasing the likelihood of occurrence, potentially though increased reproductive success as a result of an increase in high food availability during both incubation and chick rearing. Across the east of the GDR, populations on the margin of current and future emu habitat suitability may be more susceptible to extinction, particularly as they have likely undergone large past range retractions. To manage such populations, modelling population stability at smaller scales may help to detect the importance of non-climatic factors lost when modelling larger geographic scales. To better understand and manage emu populations, especially in the east of Australia, we will need a greater understanding of their biology and ecology in the wild, in particular local drivers of occurrence and abundance.

## Methods

### Species occurrence data

We predicted the potential distribution of emus over two spatial domains: the mainland of Australia (i.e. excluding Tasmania and other islands) and the Great Dividing Range and areas east of the range (‘GDR’). In terrestrial systems climate often drives distribution at global and continental scales, whereas other factors are important at smaller spatial scales^72,73^. Moreover, local adaptations may introduce differences in response to the same variables^74^. Modelling emu occurrence in the GDR may give better ecological understanding for conservation planning outcomes^74^.

We collected emu occurrence data (i.e. documentation of the occurrence of individuals at points in time and space) from the Atlas of Living Australia (ALA; http://www.ala.org.au), across Australian mainland. Tasmania, King Island and Kangaroo Island occurrences were excluded from this data, as wild emus have been absent from these island for more than a century^75,76^. The set of all downloaded presences (*n* = 83,131) was first ‘cleaned’ to remove any records with (1) a reported coordinate uncertainty of > 5 km (i.e. the observer either did not record the distance of the animal to the observer or they estimated the distance was greater than 5 km), (2) absences (i.e. locations where emus were recorded as absent, as these were biased to one geographic area), (3) fossil records (again predominately from a single region), and (4) records outside the timeframe of most predictor variables (1970–present) or records without a year. We chose points with an uncertainty of < 5 km as this is well within the area an individual may transverse regularly^43,46^, and likely represent areas still within the normal range of the emu. We did this manually and then used the *CoordinateCleaner* package in R to remove any occurrences within 10 kms of city centres, on the open ocean, in biodiversity related institutions (e.g. zoos, museums, universities) and outlier occurrences (more than 30 km from all other records)^77^. Due to the distinct appearance and vocalization of the emu, we can have a high level of certainty in the identification accuracy. After cleaning, 12,516 occurrences remained. All data manipulation and modelling were conducted using RStudio in R statistical software^78^, unless otherwise specified.

### Pseudo-absence selection and bias compensation

Much of our presence data was derived from opportunistic, rather than systematic, sightings. These may be biased to areas of high human use, for example population centres or roads^79^, and this bias increases the risk of specious correlations. To reduce this bias, we first removed duplicate presences (i.e. those with identical geographic localities) and randomly thinned data, selecting presences no less than 30 km apart. Many emus will travel widely within a 30 km radius; though they have no distinct home range^18,43,46^. Thinning to this parameter reduced the chance of repeat recordings of the same individual.

Pseudo-absences were first randomly generated in areas with low probability of presence, based on the environmental envelopes of occurrence data. To do this, we estimated a probability density kernel for presences in a principal component space - in this case based on a principal component analysis of climatic variables. Pseudo-absences were then generated with a probability complementary to the probability of a presence in that location. Pseudo-absences were then thinned to ensure that they were a minimum distance of 30 km from any presence or other pseudo-absence. To further reduce the effects of spatial bias, we generated pseudo-absences to reflect the same sampling bias as the occurrence data, following Fithian et al*.* and Molloy, Davis, Dunlop and van Etten^80,81^ i.e. there should be fewer pseudo-absences in areas with low sampling effort. We constructed a sampling bias layer using sightings of species analogous to the emu in terms of detectability and probability of reporting, i.e. large bodied vertebrates which predominantly forage on the ground, are easily recognised by non-experts, occupy a similar distributional range to the emu (i.e. across all or most states of Australia) and are reasonably common. These species are provided in Supplementary Table S3. Occurrence data for these species was downloaded from ALA and cleaned in the same manner as emu occurrence data. Remaining records were used to construct a bias layer using point density analysis in the ‘*density’* function of *spatstat* in R^82^. Selection of pseudo-absences using these methods resulted in a 1:1 ratio of presences to pseudo-absences (Aust. model: *n* = 2661:2661; GDR model: *n* = 1049:1049).

### Bioclimatic and environmental predictor variables

A suite of bioclimatic variables including eleven temperature and eight precipitation measures were downloaded from WorldClim v2.0^83^. These include average monthly climate data for the minimum, mean and maximum temperatures and for precipitation, for the years 1970–2000 at a spatial resolution of ~ 1 km^2^. The details of all bioclimatic variables can be found in Supplementary Table S1. Topographic data was also downloaded from WorldClim v2.0, derived from the Shuttle Radar Topography Mission (SRTM), and re-sampled to 1 km^2^ resolution^84,85^. As emus are known to have poor ability to conserve water, and therefore are thought to be more likely to occur near water bodies^68^, we obtained surface hydrology from GeoScience Australia and calculated a minimum distance to freshwater water per raster cell using ArcGIS 10.6^86^. Since the Australian landscape is prone to fire, and fire has been anecdotally suggested to affect the ability for emus to persist in the landscape^32^, we included the frequency at which fire occurs across each pixel. Fire frequency (1997–2009) was derived from the 2009 ‘multi-criteria analysis shell for spatial decision support data pack’^87,88^. To predict for the influence of habitat on emu occurrence, we included land use data from the ‘Australian Land Use and Management Classification v8’^88^. We estimated the proportion cover of particular agricultural land classifications relevant to emu ecology, including native grazing, modified grazing, irrigated cropping and dry cropping. For native habitats, we combined major vegetation groups from the National Vegetation Information System v5.1^89^ into rainforest, open-forest, woodland, shrubland and grassland (categorisation described in Supplementary Table S1). The emu is a generalist^19^, occupying a wide diversity of landscapes and a higher level of resolution of vegetation data was considered unlikely to be informative in understanding restrictions on emu distribution at a continental scale. As emus are thought to avoid areas of high urbanisation^23^, we included log-transformed mean human population calculated in R from gridded population estimates for years 2000, 2005, 2010 and 2020^90^ and the ‘human footprint’ index^91^ as surrogates indices of urbanisation. The ‘human footprint’ index maps cumulative human pressure, accounting for built-up environments, population density, electric power infrastructure, crop lands, pasture lands, roads, railways, and navigable waterways.

Though some degree of collinearity can be handled by machine learning methods such as the algorithms used here^92^, we performed variance inflation factor (VIF) analysis to avoid misleading results^93,94^. This was conducted using the functions *vif*, *vifcor* and *vifstep* in the R package ‘*usdm*’^93,94^. VIF indicates the degree to which the standard errors of variables are inflated due to the levels of multi-collinearity, which may skew variable importance. We calculated the VIF for all variables, using a stepwise process to exclude variables with the highest VIF, repeating this procedure until no variables with a VIF greater than 10 remained^92,95^. This process removed bio4, 5, 7, 10, 11, 12, 16 and 17 (see Supplementary Table S1 for layer details). All remaining variables were used to predict the current distribution of emus. Predictions of past and future emu distributions were made using only climatic variables, for reasons of parsimony and reliability (see “Results” above). All predictors were scaled and centred.

### Species distribution model development and evaluation

We used a model ensemble technique to overcome uncertainty implicit from variation in predictions between model types^96,97^. This allows for calculation of an ensemble prediction from a wide range of models, combining high performing models to reduce uncertainties arising from using a single algorithm^96^. We performed all modelling through BIOMOD2 v.3.1^96^ implemented in R, running six repeats of each of the nine algorithms (*n* = 54 models). We fitted and compared multiple runs of models appropriate for data with pseudo-absences: generalised linear models, general additive models, classification tree analysis, artificial neural networks, BIOCLIM, flexible discriminant analysis, multivariate adaptive regression splines, random forest, MAXENT and boosted regression trees (also known as ‘generalised boosting model’)^96^. We first performed model ‘tuning’ to optimize parameters, determining optimal model complexity, to increase the predictive ability of models and reduce problems of over fitting^98^.

We established variable importance to each model by using a permutation method, which randomises each variable individually and then projects the model with the randomised variable, keeping all other variables unchanged. The model, with the randomised variable, is then correlated with those of the original model. Variable importance for each predictor is then calculated as one minus the correlation, as per Thuiller et al*.*^96^. Here, higher values indicate predictors that are more important for the model. In order to evaluate models on data separate from that used to calibrate the models, we used 70% of the data, sampled randomly. Models were evaluated by calculating the area under the curve (AUC) of the receiver operating characteristics curve (ROC)^99^, Kappa statistic and the true skill statistics (TSS)^100^. To create an ensemble model for current emu distribution, we used models from each run of each algorithm type with an AUC of ≥ 0.9 (range 0–1, with values equal to or less than 0.5 predicting no better than random), a TSS of ≥ 0.7 (range − 1 to + 1, with values equal to or less than zero predicting no better than random), and a Kappa of ≥ 0.7 (range 0–1; perfect fit = 1). These thresholds are generally considered to represent models with high accuracy^100^. We then took models that met the accuracy threshold to create ensemble predictions, which were weighted averages of the predicted habitat suitability across algorithms, weighted by TSS. We used TSS rather than AUC, as these are intended for use with true absence data (rather than pseudo-absence data), and thus may be somewhat inflated here by the use of pseudo-absence data^101^. By weighting models by TSS, models with higher predictive power had greater influence on the final ensemble. We additionally measured ensemble model performance using the Boyce Index, which indicates how much predictions differ from a random distribution of the observed presences across the prediction gradient^102^. The index is continuous between − 1 and + 1, with positive values indicating the model prediction is consistent with the distribution of presences and negative values indicating poorly predicting models (zero being model predictions no different from random)^103^. All model performance values are given as the average ± the standard deviation.

### Past and future climatic models

We used the results of the current distribution climate-only ensembles to hindcast and forecast emu distributions, i.e. to estimate the past and future potential distributions of emus given changes in climate. To perform future and past predictions of distribution, we used global climatic models downloaded from WorldClim 1.4, which were downscaled and calibrated (bias corrected) using ‘current’ climate baselines. We used the coupled model inter-comparison project (CMIP5) to predict future climate, i.e. the Community Climate System Model (CCSM4)^104^. The CCSM4 is available for both future and past climate scenarios, which provides for direct comparison. For predictions of future emu distribution, we first created models using scenarios which included minimal reductions in emissions (the most conservative representative concentration pathway, 8.5). As the effect of severe climate change in emus was found to be slight, we did not analyse the effects of scenarios with larger reductions in emissions. To estimate emu distribution in the past, we used CCSM4 mid-Holocene scenarios (~ 6000 ya)^105,106^. This represents a time prior to the influence of European settlement and large environmental changes in Australia, and is relatively recent in terms of the speciation of emus from other ratites (~ 31 mya^107,108^). Reliable climate data is difficult to obtain for times immediately prior to European settlement, and environmental mapping is rarely conducted for these times. As such, this time period represents the most robust way of estimating historical distribution. For these projections, we used the same subset of bioclimatic variables described above. To validate our predictions, we also modelled using the Commonwealth Scientific and Industrial Research Organization (CSIRO) ACCESS 1.0, which uses the most reliable meteorological data for Australia, but for which only future models are available. We present these future climate distribution models in our supplementary material for comparison.

To estimate the change in emu range over time, the simulated past, future and current distribution ensembles were converted to binary (presence/absence) outputs by selecting the threshold that maximized the TSS score. We did this simplifying step here only to avoid unnecessary loss of information and assumptions created by binary predictions^108^. We compared the number of cells occupied under each time period and calculated the percentage decrease or increase in range. The ability of a species to alter its range in response to climate change is mediated by the dispersal capacity of the species. A reliable estimation of dispersal capability of emus across Australia is not available, with dispersal distances only available for certain populations^47^. We therefore examined two extreme scenarios as estimates of the upper and lower limits of range changes; firstly, unlimited dispersal where the entire projection of future range is considered to be the actual range and secondly, limited/no dispersal where the future distribution results from the overlap between current and projected future range. This was done using both the Australian mainland and the GDR-only models.

As our future and past climate models potentially made predictions based on novel climate conditions, we used a ‘multivariate environmental similarity surface’ (MESS) analysis to identify the degree of extrapolation for each climate model (i.e. areas with environments outside the range represented in the current climate from occurrence points used in our model)^109^. We use the *mess* function in the *dismo* package^109^ to identify and map areas and predictors that should be taken with caution when interpreting model outputs. This provides a map for each climate parameter and an aggregate map across localities and climate parameter sets using the MESS statistic, with positive values indicating cells that are similar to the environmental values used for models, whereas negative values indicate novel climate. We also explore changes in correlations between climate variables for each climate model using Pearson’s correlation coefficient^109^. The outputs of both methods are presented in the Supplementary Material. All maps were created using R.

## Supplementary Information

Supplementary Information

## Data Availability

R script for modeling and occurence data (not taken directly from those sources listed in Supplementary Table S1) is available for download through Dryad Doi https://doi.org/10.5061/dryad.tht76hdx1.
