# Supplementary material for: Past and future potential range changes in one of the last large vertebrates of the Australian continent, the emu Dromaius novaehollandiae
Source: Sci Rep. 2021 Jan 13;11:851. doi: 10.1038/s41598-020-79551-0 (PMC7807066; doi:10.1038/s41598-020-79551-0)

Past and future potential range changes in one of the last large vertebrates of the Australian continent, the emu *Dromaius novaehollandiae*

**Julia Ryeland^1*^, Tristan T. Derham^2^, Ricky J. Spencer^1^**

^1^ Hawkesbury Institute for the Environment, Western Sydney University, Hawkesbury Campus, Locked Bag 1797, Penrith, NSW, Australia.

^2^ School of Natural Sciences, University of Tasmania, Private Bag 55, Hobart 7001, Australia

***Corresponding author:**
Julia Ryeland,
Email: Julia.ryeland@outlook.com
Phone number: +61 434408460
Address: Hawkesbury Institute for the Environment
Locked bag: 1797, PENRITH, NSW, 2751, AUST.

## Number of figures: 4 Number of tables: 1 Supplementary information: 8 Competing interests: The authors declare no competing interests

**Supplementary Table S1** Climatic and environmental factors used in analyses include all variables. Variables with an * are those that were included in climate only models

| **Name** | **Description** | **Source** |
| --- | --- | --- |
| *Climatic variables* | | |
| **B01 *** | Annual Mean Temperature | WorldClim (www.worldclim.com) |
| **B02 *** | Mean Diurnal Range (Mean of monthly (max temp min temp)) |  |
| **B03 *** | Isothermality (BIO2/BIO7) (* 100) |  |
| **B04** | Temperature Seasonality (standard deviation *100) |  |
| **B05** | Max Temperature of Warmest Month |  |
| **B06 *** | Min Temperature of Coldest Month |  |
| **B07** | Temperature Annual Range (BIO5-BIO6) |  |
| **B08 *** | Mean Temperature of Wettest Quarter |  |
| **B09 *** | Mean Temperature of Driest Quarter |  |
| **B10** | Mean Temperature of Warmest Quarter |  |
| **B11** | Mean Temperature of Coldest Quarter |  |
| **B12** | Annual Precipitation |  |
| **B13** | Precipitation of Wettest Month |  |
| **B14 *** | Precipitation of Driest Month |  |
| **B15 *** | Precipitation Seasonality (Coefficient of Variation) |  |
| **B16** | Precipitation of Wettest Quarter |  |
| **B17** | Precipitation of Driest Quarter |  |
| **B18 *** | Precipitation of Warmest Quarter |  |
| **B19 *** | Precipitation of Coldest Quarter |  |
| *Environmental variables* | | |
| **Elevation** | Shuttle Radar Topography Mission (SRTM) altitude | WorldClim ([www.worldclim.com](http://www.worldclim.com)) |
| **Rainforest** | Rainforests and vine thickets | NVIS (v5.0) veg MVG 1 ([https://www.environment.gov.au](https://www.environment.gov.au/)) |
| **Open forest** | Eucalypt tall open forests, Eucalypt open forests and Eucalypt low open forests | NVIS (v5.0) veg MVG 2 – 4 ([https://www.environment.gov.au](https://www.environment.gov.au/)) |
| **Woodland** | Eucalypt woodlands, Acacia forests and woodlands, Callitris forests and woodlands, Casuarina forests and woodlands, Melaleuca forests and woodlands, Other forests and woodlands, Eucalypt open woodlands, Tropical eucalypt woodlands/grasslands, Acacia open woodlands, Mallee woodlands and shrublands, Mallee open woodlands and sparse mallee shrublands & Other open woodlands | NVIS (v5.0) veg MVG 5 – 14 & 31 – 32 [https://www.environment.gov.au](https://www.environment.gov.au/) |
| **Scrubland** | Low closed forests and tall closed shrublands, Acacia shrublands, Other shrublands & Heathlands | NVIS (v5.0) veg MVG 15 – 18 & 22 [https://www.environment.gov.au](https://www.environment.gov.au/) |
| **Grassland** | Tussock grasslands, Hummock grasslands, Other grasslands, herblands, sedgelands and rushlands & Chenopod shrublands, samphire shrublands and forblands | NVIS (v5.0) veg MVG 19 – 21  [https://www.environment.gov.au](https://www.environment.gov.au/) |
| **Farm land– dry cropping** | Land that is primarily used for cropping with a dryland farming systems. This may include a rotating crop system, with area under pasture for some of the time | Australian Land Use and Management Classification (v8) [**https://www.agriculture.gov.au**](https://www.agriculture.gov.au/abares/aclump/land-use/alum-classification) |
| **Farm land- irrigation** | Agricultural land uses where water is applied to promote additional growth over normally dry periods | Australian Land Use and Management Classification (v8) [https://www.agriculture.gov.au](https://www.agriculture.gov.au/abares/aclump/land-use/alum-classification) |
| **Farm land – modified grazing** | Pasture and forage production, both annual and perennial, with significant active modification or replacement of the initial vegetation with greater than 50 per cent dominant exotic species | Australian Land Use and Management Classification (v8) [https://www.agriculture.gov.au](https://www.agriculture.gov.au/abares/aclump/land-use/alum-classification) |
| **Farm land – native grazing** | Land with grazing by domestic stock on native vegetation where there has been limited or no deliberate attempt at pasture modification. Some change in species composition may have occurred. | Australian Land Use and Management Classification (v8) [https://www.agriculture.gov.au](https://www.agriculture.gov.au/abares/aclump/land-use/alum-classification) |
| **Distance to freshwater** | Distance to any freshwater calculated from surface hydrology polygons- regional scale’ layer | Geoscience Australia ([https://www.ga.gov.au](https://www.ga.gov.au/)) |
| **Fire frequency** | Number of times a cell has been burnt (1988-2015 data) | Bureau of Agricultural and Resource Economics and Sciences (http://agriculture.gov.au/abares) |
| **Distance from roads** | Distance calculated from state combined road centerline data (used as a bias layer only) | Individual state government bodies (on request) |
| **Population** | Gridded Population of the World, v4 (GPWv4) 2017 (Years 2000, 2005, 2010, 2015, 2020) | NASA Earthdata (<https://earthdata.nasa.gov/>) |
| **Human footprint** | Cumulative human pressure on the environment in 2009, at a spatial resolution of ~1 km. The human pressure is measured using eight variables including built-up environments, population density, electric power infrastructure, crop lands, pasture lands, roads, railways, and navigable waterways. | NASA Socio economic data and application centre <https://sedac.ciesin.columbia.edu/> |

**Supplementary Table S2** Model performance statistics for each run of each algorithm type at both domains, with all variables and with only bioclimatic variables. *n* is the product of the number of algorithms and the number of model runs per algorithm.

| **Domain** | **Variables** | **Algorithm** | **Area under the ROC Curve (AUC)** | | | | | | **True Skill Statistic (TSS)** | | | | | | **KAPPA** | | | | | | ***n*** |
| --- | --- | --- | --- | --- | --- | --- | --- | --- | --- | --- | --- | --- | --- | --- | --- | --- | --- | --- | --- | --- | --- |
|  |  |  | Run 1 | Run 2 | Run 3 | Run 4 | Run 5 | Run 6 | Run 1 | Run 2 | Run 3 | Run 4 | Run 5 | Run 6 | Run 1 | Run 2 | Run 3 | Run 4 | Run 5 | Run 6 |  |
| **Australia-wide** | **All** | GLM | **0.92** | **0.92** | **0.91** | **0.91** | **0.93** | **0.91** | **0.73** | **0.72** | **0.71** | **0.73** | **0.75** | **0.7** | **0.73** | **0.72** | **0.71** | **0.73** | **0.75** | **0.7** | 6 |
|  |  | GBM | **0.94** | **0.94** | **0.94** | **0.94** | **0.95** | **0.94** | **0.77** | **0.77** | **0.77** | **0.77** | **0.77** | **0.76** | **0.77** | **0.77** | **0.77** | **0.77** | **0.77** | **0.76** | 6 |
|  |  | GAM | **0.94** | **0.93** | **0.93** | **0.94** | **0.95** | **0.94** | **0.77** | **0.77** | **0.75** | **0.77** | **0.79** | **0.75** | **0.77** | **0.77** | **0.75** | **0.77** | **0.79** | **0.75** | 6 |
|  |  | RF | **0.97** | **0.97** | **0.97** | **0.97** | **0.97** | **0.97** | **0.83** | **0.83** | **0.84** | **0.83** | **0.86** | **0.83** | **0.83** | **0.83** | **0.84** | **0.83** | **0.86** | **0.83** | 6 |
|  |  | MARS | **0.91** | **0.91** | **0.91** | **0.91** | **0.92** | **0.91** | **0.72** | **0.71** | **0.7** | **0.72** | **0.73** | **0.7** | **0.72** | **0.71** | **0.7** | **0.72** | **0.73** | **0.7** | 6 |
|  |  | CTA | **0.91** | **0.91** | **0.9** | **0.9** | **0.92** | **0.91** | **0.75** | **0.75** | **0.77** | **0.77** | **0.76** | **0.77** | **0.75** | **0.75** | **0.77** | **0.77** | **0.76** | **0.77** | 6 |
|  |  | SRE | 0.68 | 0.66 | 0.67 | 0.68 | 0.68 | 0.67 | 0.36 | 0.33 | 0.35 | 0.36 | 0.37 | 0.34 | 0.36 | 0.32 | 0.34 | 0.36 | 0.37 | 0.34 | 0 |
|  |  | FDA | 0.89 | 0.89 | 0.89 | 0.89 | 0.9 | 0.89 | 0.67 | 0.67 | 0.65 | 0.68 | 0.68 | 0.64 | 0.67 | 0.67 | 0.65 | 0.68 | 0.68 | 0.64 | 0 |
|  |  | MAXENT | 0.49 | 0.5 | 0.5 | 0.5 | 0.49 | 0.49 | 0 | 0.01 | 0 | 0 | 0 | 0 | 0 | 0.01 | 0 | 0 | 0 | 0 | 0 |
|  | **Total: 36** | | | | | | | | | | | | | | | | | | | | |
|  | **Bioclimatic-only** | GAM | **0.93** | **0.93** | **0.93** | **0.93** | **0.92** | **0.93** | **0.75** | **0.73** | **0.74** | **0.74** | **0.72** | **0.74** | **0.75** | **0.73** | **0.74** | **0.74** | **0.72** | **0.74** | 6 |
|  |  | GLM | **0.91** | **0.91** | **0.91** | **0.92** | **0.9** | **0.91** | **0.73** | **0.72** | **0.72** | **0.73** | **0.69** | **0.72** | **0.73** | **0.72** | **0.72** | **0.73** | **0.69** | **0.72** | 6 |
|  |  | GBM | **0.94** | **0.94** | **0.93** | **0.94** | **0.93** | **0.93** | **0.76** | **0.77** | **0.75** | **0.76** | **0.74** | **0.75** | **0.76** | **0.77** | **0.75** | **0.76** | **0.74** | **0.75** | 6 |
|  |  | RF | **0.96** | **0.97** | **0.96** | **0.97** | **0.96** | **0.96** | **0.83** | **0.83** | **0.84** | **0.83** | **0.82** | **0.82** | **0.83** | **0.83** | **0.84** | **0.83** | **0.82** | **0.82** | 6 |
|  |  | MARS | **0.91** | **0.91** | **0.91** | **0.92** | **0.9** | **0.91** | **0.71** | **0.7** | **0.71** | **0.72** | **0.68** | **0.71** | **0.71** | **0.7** | **0.71** | **0.72** | **0.68** | **0.71** | 6 |
|  |  | CTA | **0.89** | **0.91** | **0.91** | **0.9** | **0.91** | **0.92** | **0.75** | **0.74** | **0.76** | **0.76** | **0.76** | **0.77** | **0.75** | **0.74** | **0.76** | **0.76** | **0.76** | **0.77** | 6 |
|  |  | SRE | 0.7 | 0.72 | 0.72 | 0.72 | 0.72 | 0.72 | 0.41 | 0.45 | 0.44 | 0.44 | 0.44 | 0.45 | 0.41 | 0.45 | 0.44 | 0.44 | 0.44 | 0.45 | 0 |
|  |  | FDA | **0.91** | 0.9 | **0.9** | **0.91** | 0.89 | 0.9 | **0.72** | 0.69 | **0.7** | **0.73** | 0.67 | 0.69 | 0.72 | 0.69 | 0.7 | 0.73 | 0.67 | 0.69 | 3 |
|  |  | MAXENT | 0.9 | 0.89 | 0.89 | 0.91 | 0.89 | 0.89 | 0.67 | 0.63 | 0.62 | 0.66 | 0.62 | 0.65 | 0.67 | 0.63 | 0.62 | 0.66 | 0.62 | 0.65 | 0 |
|  | **Total: 39** | | | | | | | | | | | | | | | | | | | | |
| **Great Dividing Range** | **All** | GLM | **0.93** | **0.92** | **0.92** | 0.91 | 0.90 | **0.92** | **0.74** | **0.71** | **0.71** | 0.69 | 0.68 | **0.70** | 0.75 | 0.71 | 0.71 | 0.69 | 0.68 | 0.7 | 4 |
|  |  | GBM | **0.94** | **0.93** | **0.94** | **0.93** | **0.93** | **0.93** | **0.75** | **0.72** | **0.74** | **0.70** | **0.71** | **0.71** | **0.75** | **0.72** | **0.74** | **0.7** | **0.71** | **0.71** | 6 |
|  |  | GAM | **0.94** | **0.94** | **0.94** | **0.93** | **0.91** | **0.92** | **0.76** | **0.73** | **0.77** | **0.75** | **0.71** | **0.73** | **0.76** | **0.73** | **0.77** | **0.75** | **0.71** | **0.73** | 6 |
|  |  | RF | **0.97** | **0.96** | **0.97** | **0.96** | **0.96** | **0.96** | **0.8** | **0.81** | **0.81** | **0.8** | **0.81** | **0.8** | **0.8** | **0.81** | **0.81** | **0.8** | **0.81** | **0.8** | 6 |
|  |  | MARS | 0.92 | **0.93** | **0.92** | 0.91 | **0.91** | **0.91** | 0.67 | **0.7** | **0.71** | 0.66 | **0.7** | **0.67** | 0.67 | **0.7** | **0.71** | 0.66 | **0.7** | **0.67** | 4 |
|  |  | CTA | 0.87 | 0.87 | 0.89 | 0.87 | 0.89 | 0.87 | 0.65 | 0.65 | 0.71 | 0.66 | 0.65 | 0.65 | 0.65 | 0.66 | 0.71 | 0.66 | 0.65 | 0.65 | 0 |
|  |  | SRE | 0.58 | 0.61 | 0.64 | 0.61 | 0.63 | 0.63 | 0.17 | 0.21 | 0.28 | 0.22 | 0.26 | 0.26 | 0.17 | 0.21 | 0.28 | 0.22 | 0.26 | 0.26 | 0 |
|  |  | FDA | **0.93** | **0.92** | **0.93** | **0.92** | **0.91** | **0.92** | **0.72** | **0.7** | **0.75** | **0.73** | **0.69** | **0.72** | **0.72** | **0.7** | **0.75** | **0.73** | **0.69** | **0.72** | 6 |
|  |  | MAXENT | 0.63 | 0.62 | 0.63 | 0.62 | 0.66 | 0.62 | 0.45 | 0.44 | 0.44 | 0.45 | 0.44 | 0.44 | 0.66 | 0.64 | 0.66 | 0.66 | 0.66 | 0.69 | 0 |
|  | **Total: 32** | | | | | | | | | | | | | | | | | | | | |
|  | **Bioclimatic-only** | GAM | **0.92** | **0.93** | **0.92** | **0.94** | **0.92** | **0.93** | **0.74** | **0.72** | **0.72** | **0.76** | **0.74** | **0.77** | **0.74** | **0.72** | **0.72** | **0.76** | **0.74** | **0.77** | 6 |
|  |  | GLM | 0.88 | 0.89 | 0.9 | 0.91 | **0.91** | 0.9 | 0.64 | 0.63 | 0.65 | 0.68 | **0.70** | 0.66 | 0.64 | 0.63 | 0.65 | 0.68 | **0.70** | 0.66 | 1 |
|  |  | GBM | 0.91 | 0.92 | 0.92 | **0.93** | **0.94** | **0.94** | 0.68 | 0.68 | 0.69 | **0.71** | **0.77** | **0.73** | 0.68 | 0.68 | 0.69 | **0.71** | **0.77** | **0.73** | 3 |
|  |  | RF | **0.95** | **0.96** | **0.96** | **0.96** | **0.97** | **0.96** | **0.79** | **0.78** | **0.79** | **0.79** | **0.82** | **0.81** | **0.79** | **0.78** | **0.79** | **0.79** | **0.82** | **0.81** | 6 |
|  |  | MARS | 0.88 | 0.88 | **0.91** | **0.92** | 0.91 | 0.91 | 0.61 | 0.62 | **0.71** | **0.71** | 0.69 | 0.69 | 0.61 | 0.62 | **0.71** | 0.71 | 0.69 | **0.69** | 2 |
|  |  | CTA | **0.88** | 0.89 | 0.87 | **0.92** | **0.92** | **0.92** | **0.72** | 0.66 | 0.68 | **0.70** | **0.75** | **0.74** | 0.72 | 0.66 | 0.68 | 0.71 | 0.75 | 0.74 | 4 |
|  |  | SRE | 0.59 | 0.59 | 0.59 | 0.63 | 0.62 | 0.61 | 0.19 | 0.19 | 0.18 | 0.2 | 0.22 | 0.22 | 0.19 | 0.19 | 0.18 | 0.2 | 0.22 | 0.22 | 0 |
|  |  | FDA | 0.88 | 0.88 | 0.89 | 0.91 | 0.9 | 0.89 | 0.64 | 0.59 | 0.64 | 0.68 | 0.68 | 0.66 | 0.64 | 0.59 | 0.64 | 0.68 | 0.68 | 0.66 | 0 |
|  |  | MAXENT | 0.63 | 0.62 | 0.62 | 0.63 | 0.62 | 0.62 | 0.32 | 0.45 | 0.44 | 0.44 | 0.44 | 0.44 | 0.21 | 0.66 | 0.64 | 0.66 | 0.64 | 0.65 | 0 |
|  | **Total: 22** | | | | | | | | | | | | | | | | | | | | |

**Supplementary Table S3** Substitute species used to create a bias layer reflecting sampling effort across Australia

| Common name | Scientific name | Included occurrences |
| --- | --- | --- |
| **Magpie goose** | *Anseranas semipalmata* | 49,115 |
| **White-necked heron** | *Ardea pacifica* | 28,415 |
| **Sulphur-crested cockatoo** | *Cacatua galerita* | 86,443 |
| **Australian wood duck** | *Chenonetta jubata* | 38,190 |
| **Australian raven** | *Corvus coronoides* | 53,091 |
| **Black swan** | *Cygnus atratus* | 16,229 |
| **Pink galah** | *Eolophus roseicapilla* | 47,792 |
| **Brolga** | *Grus rubicunda* | 5,297 |
| **Australian magpie** | *Gymnorhina tibicen* | 66,384 |
| **Western grey kangaroo** | *Macropus fuliginosus* | 12,911 |
| **Eastern grey kangaroo** | *Macropus giganteus* | 20,006 |
| **Common walaroo** | *Osphranter robustus* | 12,079 |
| **Red kangaroo** | *Osphranter rufus* | 14,932 |
| **Australian pelican** | *Pelecanus conspicillatus* | 9,411 |
| **Australian white ibis** | *Threskiornis moluccus* | 9,949 |
| **Straw-necked ibis** | *Threskiornis spinicollis* | 4,058 |
|  | ***Total*** | **474, 302** |

**Supplementary Figure S1** Predicted probability of emu occurrence from ensemble models across the Australian mainland and the Great Dividing Range region at 2050 CE with predictions from the CSIRO ACCESS 2.0 climate model, bioclimatic variables only. Maps were created through R statistical software [78]. The figures were generated in R (version 3.6.0, https://www.r-project.org/).


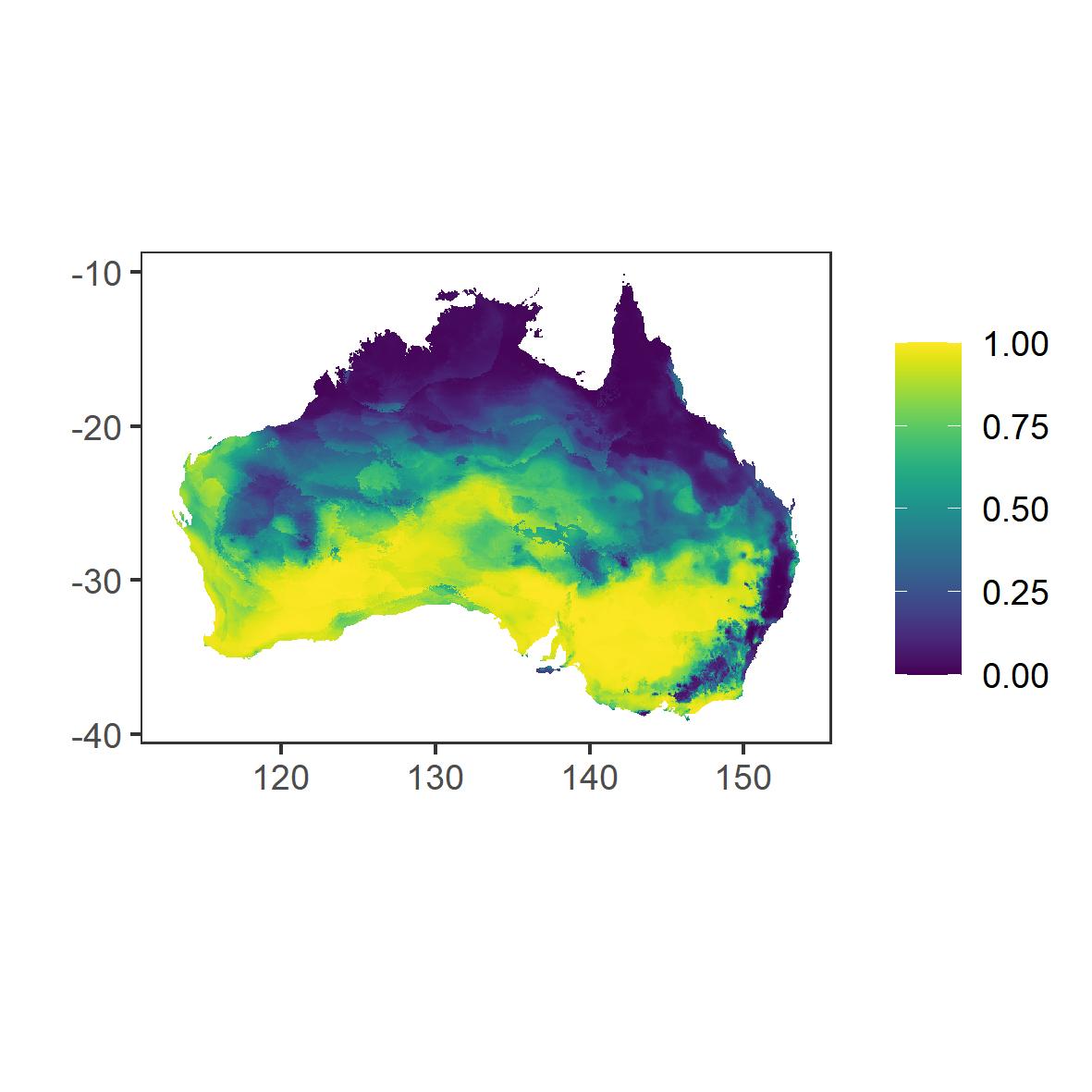

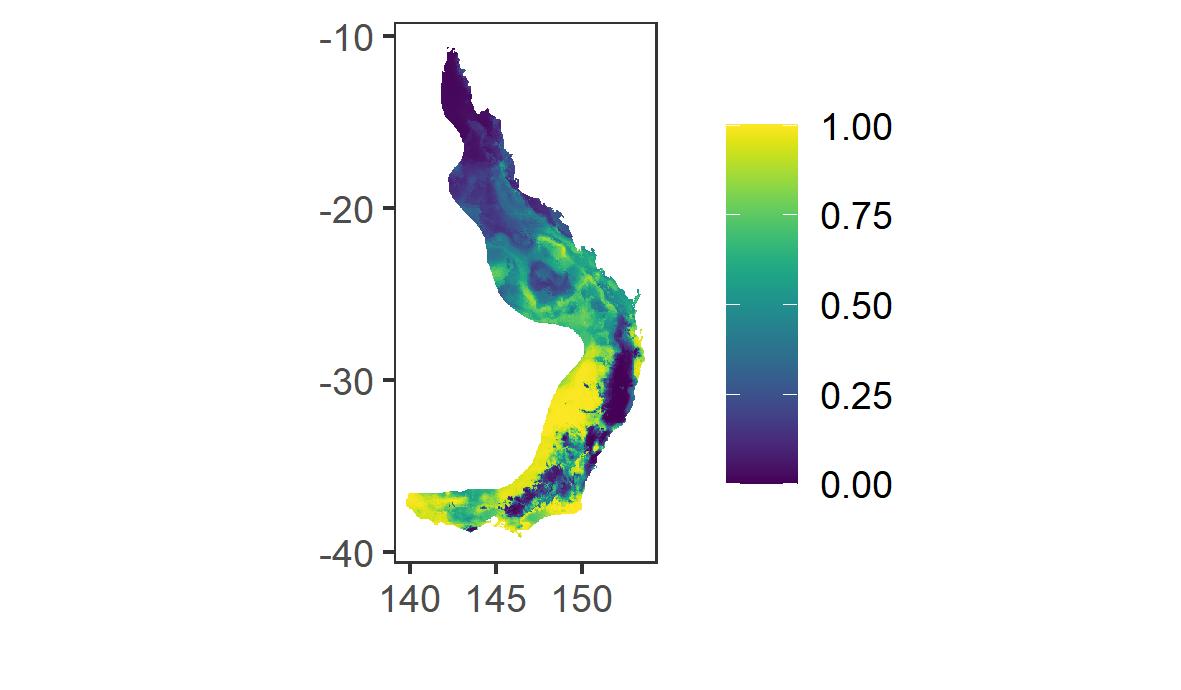

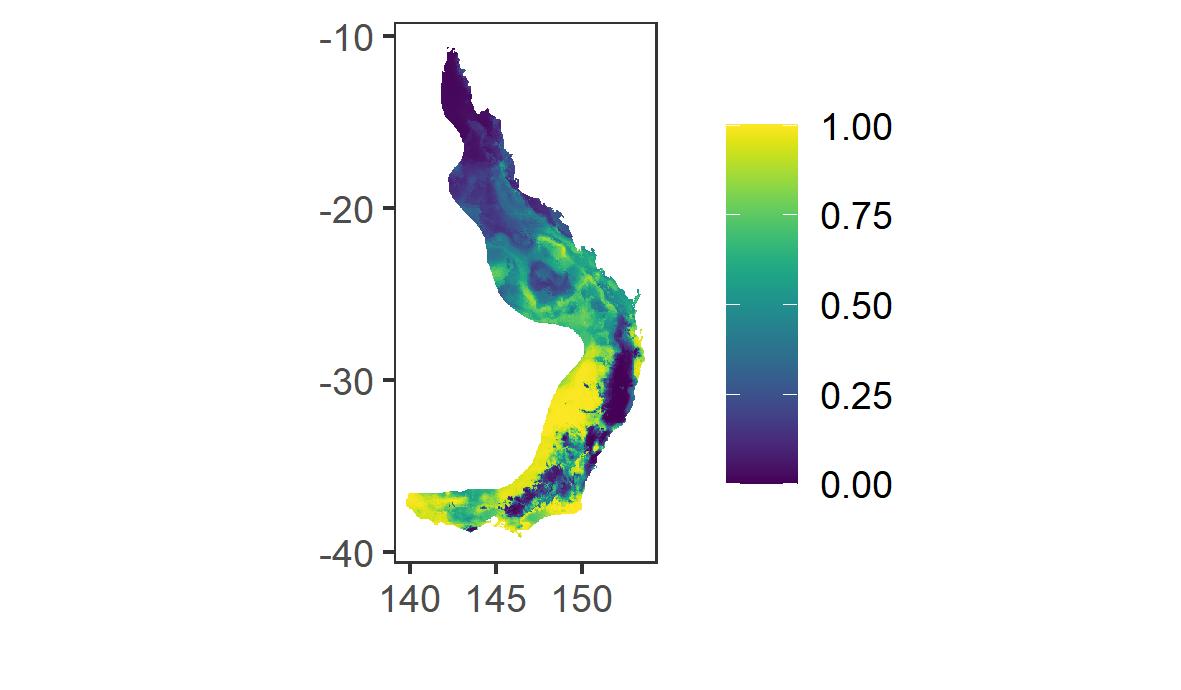


*Australia wide*

*Great Dividing Range*

**Supplementary Figure S2** Predicted distribution change from the present to 2070 CE, using CSIRO ACCESS 2.0 climate predictions, across Australia and across the Great Dividing Range. Dispersal is assumed in calculating difference. Maps were created through R statistical software [78]. The figures were generated in R (version 3.6.0, https://www.r-project.org/).


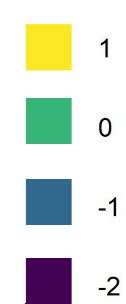


Loss

Suitable and stable

Unsuitable and stable

Gain


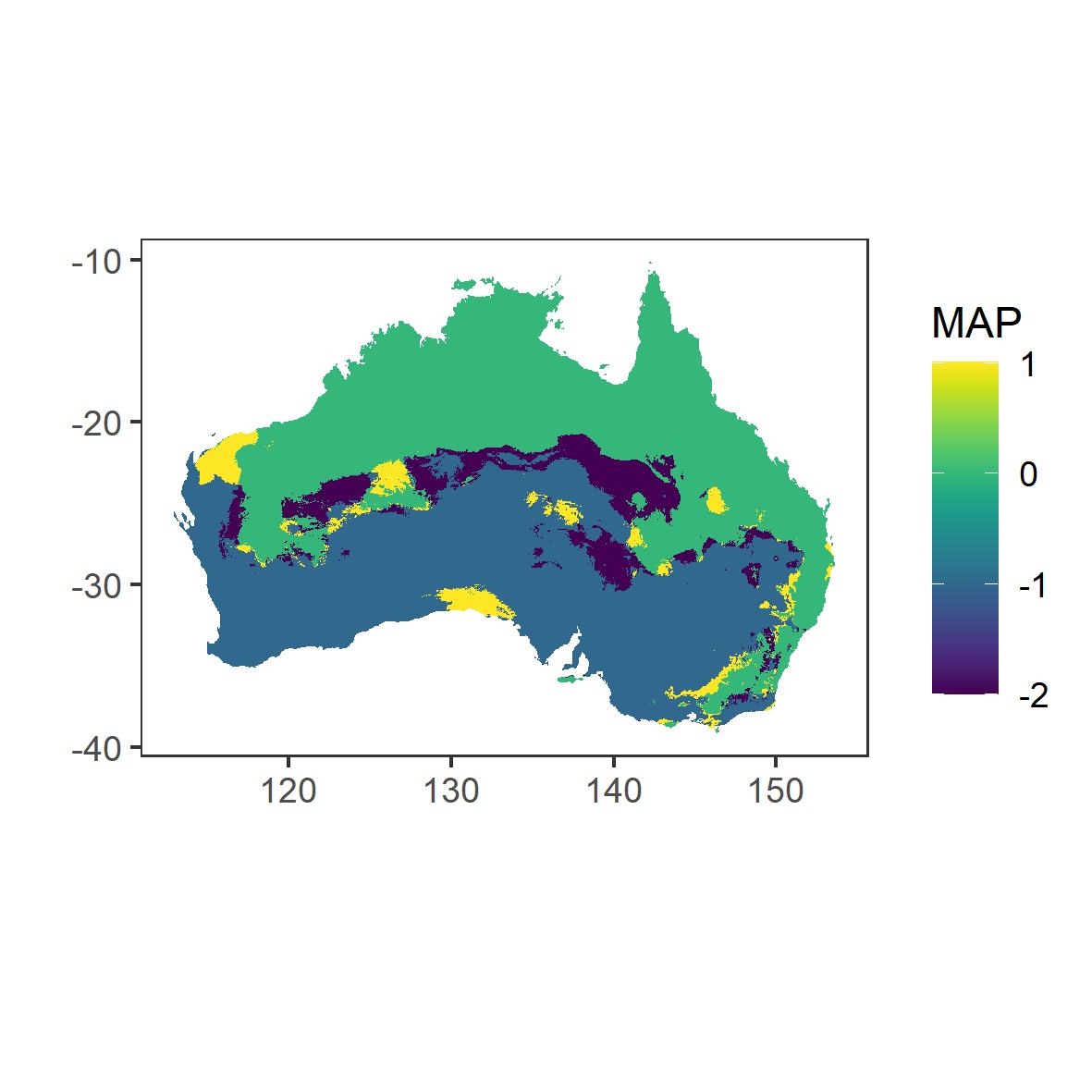

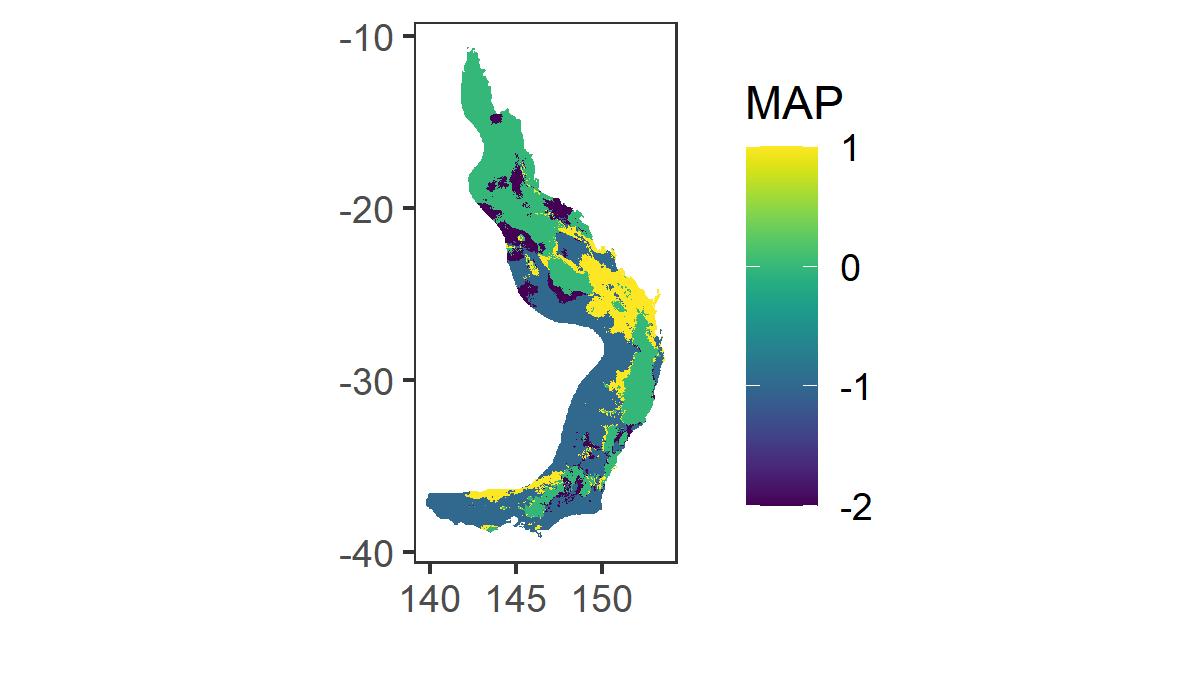


**Supplementary Figure S3** Coefficient of variation (SD/mean) for individual models included in weighted bioclimatic ensembles. Lower values represent lower uncertainty in observed range, and a better model fit. Maps were created through R statistical software [78]. The figures were generated in R (version 3.6.0, https://www.r-project.org/).

*Great Dividing Range*

*Australia wide*

*Past*

*Current*

*Future (CCSM4)*

*Future (ACCESS)*


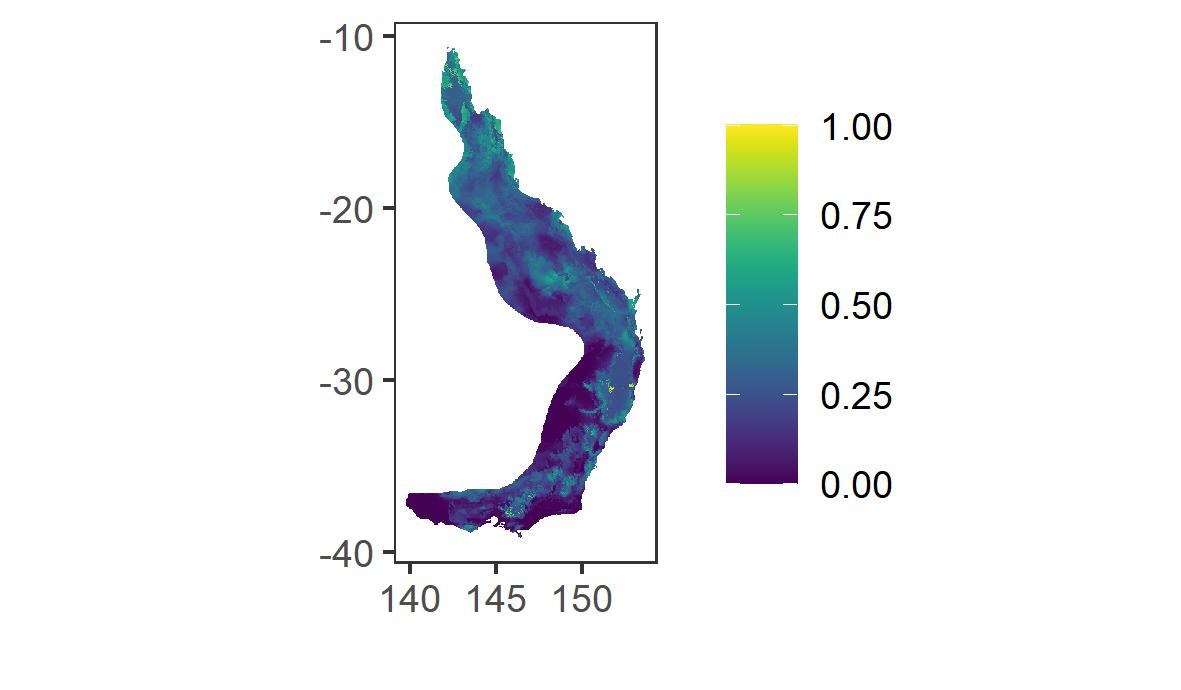

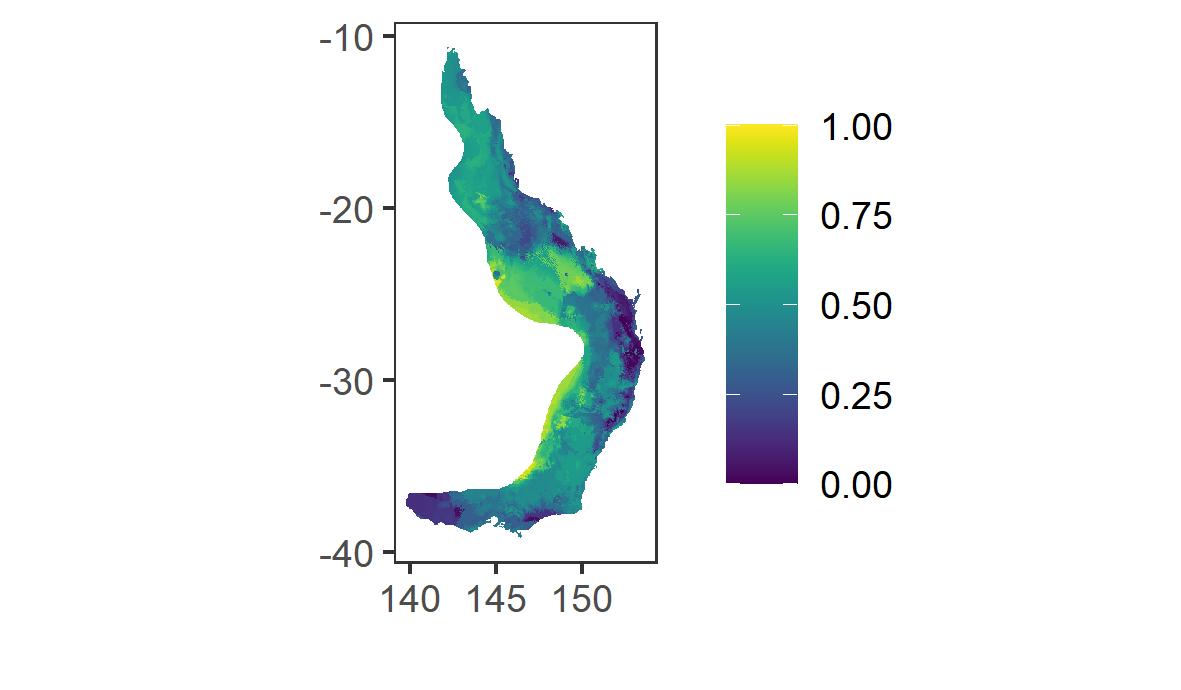

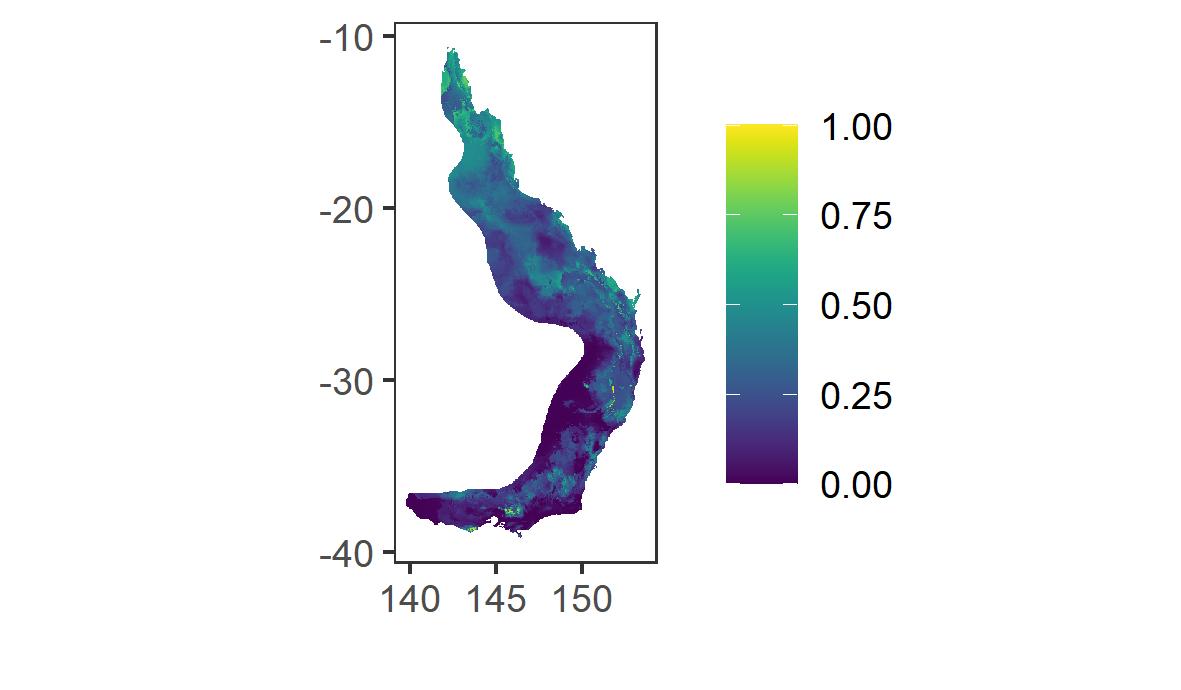

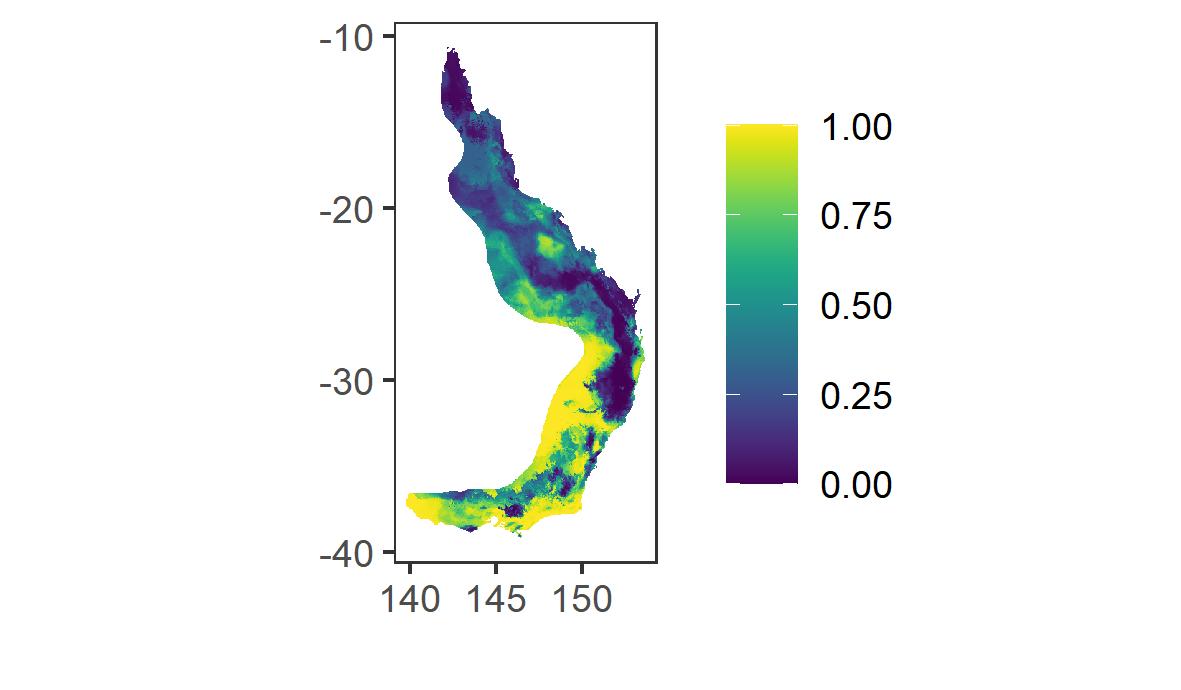

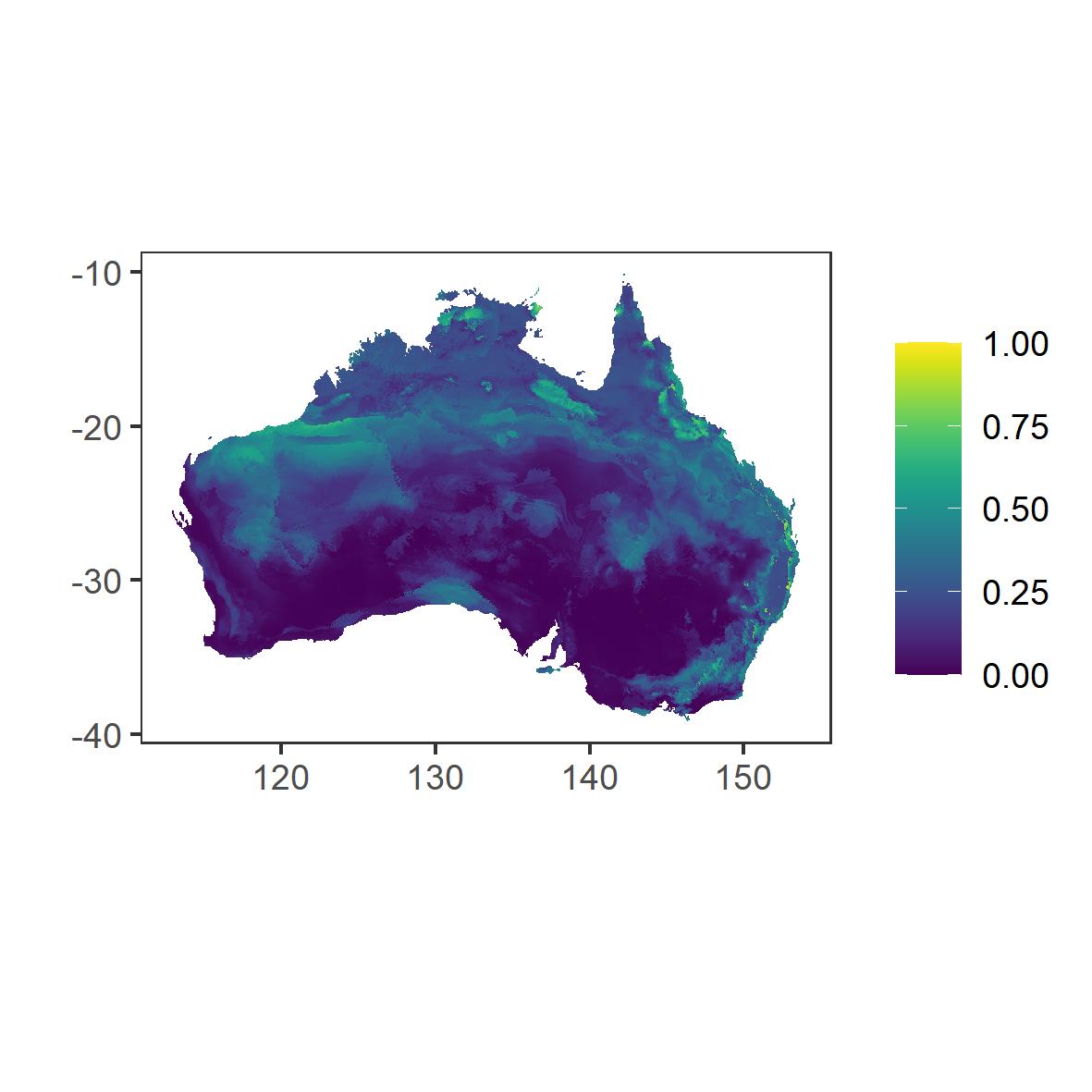

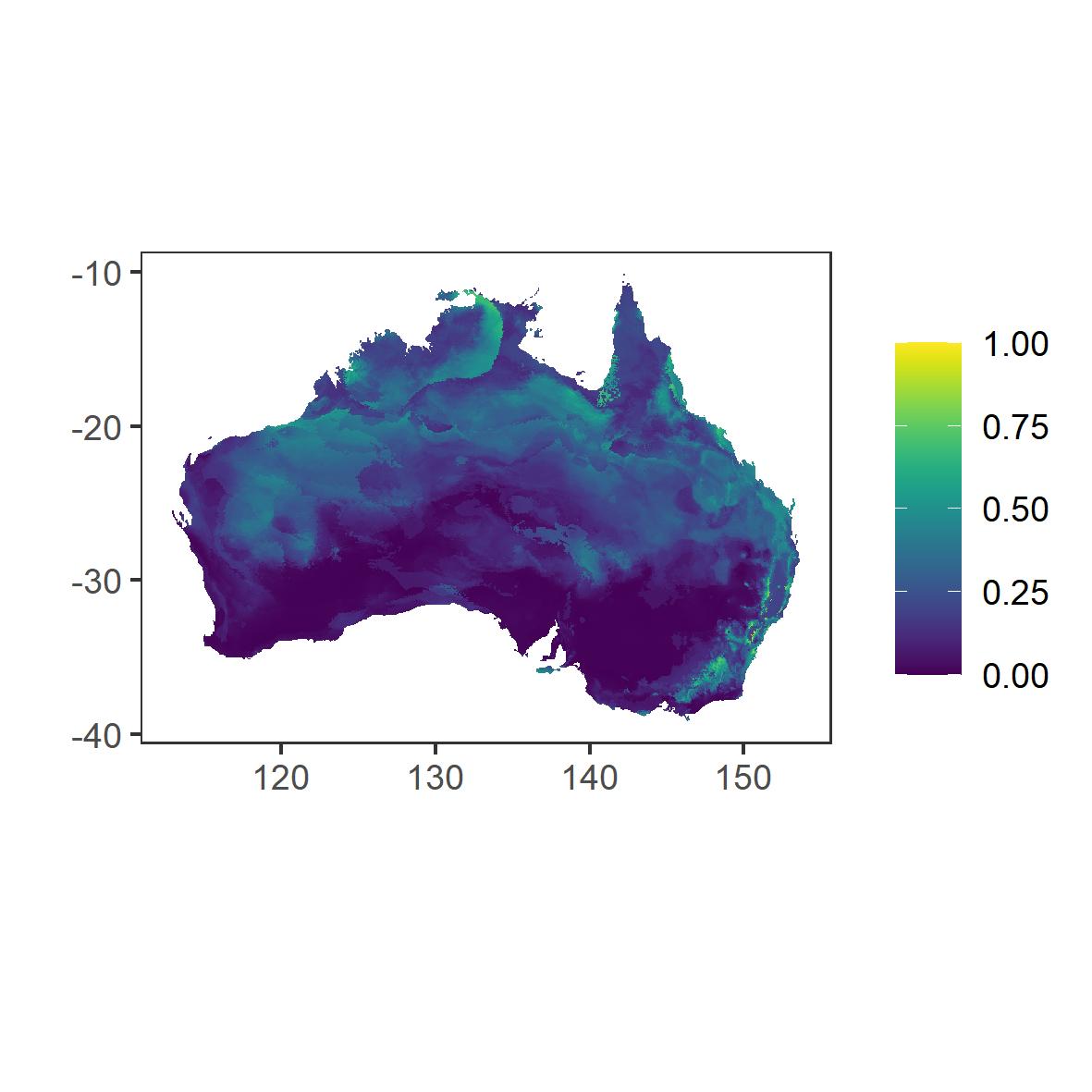

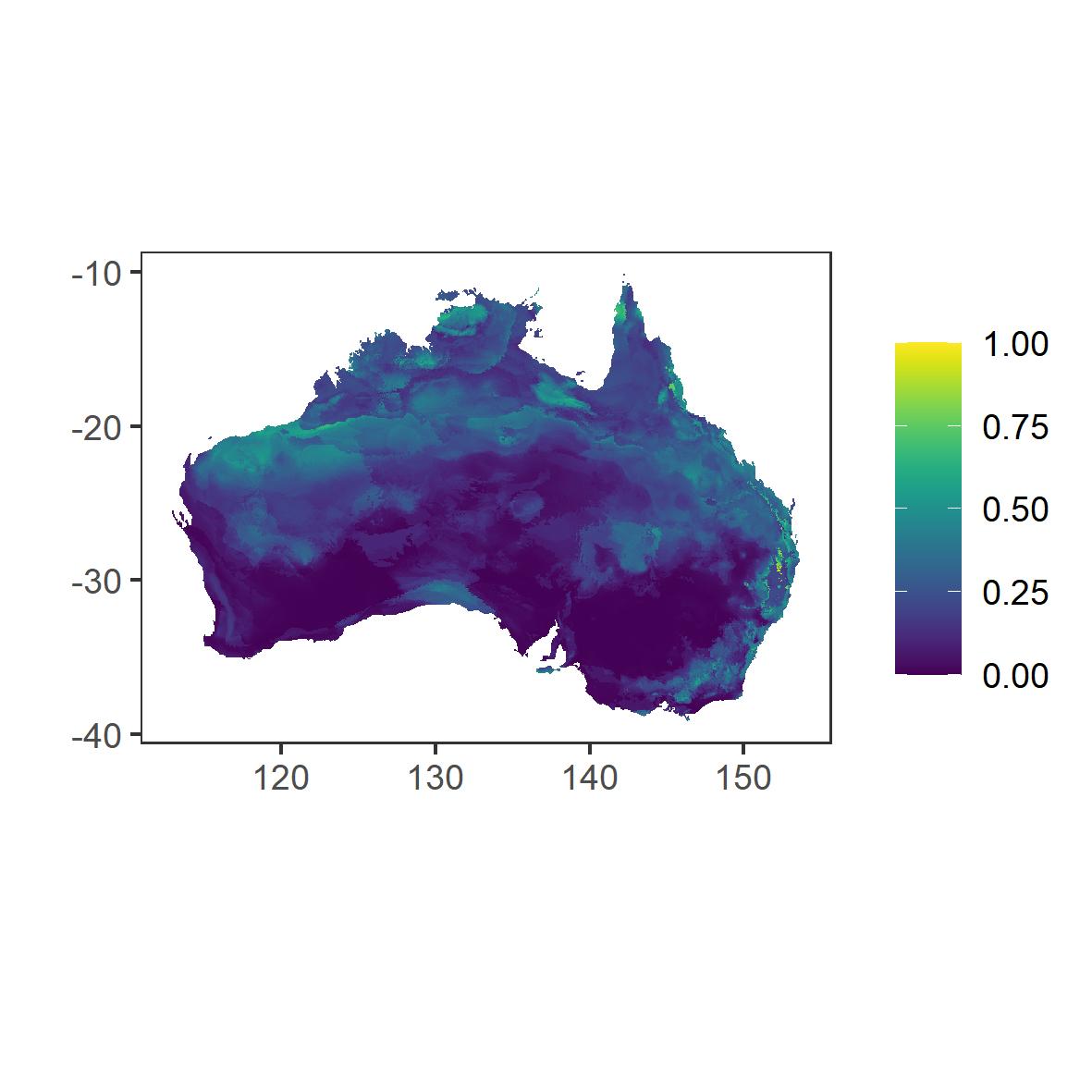

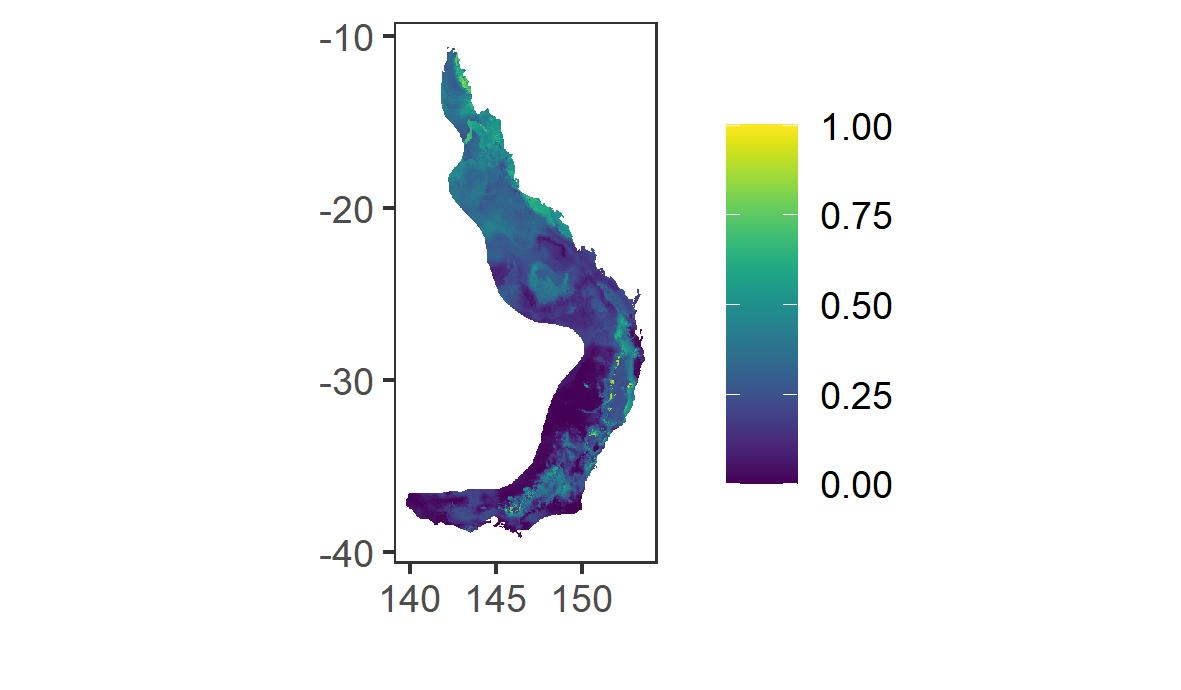

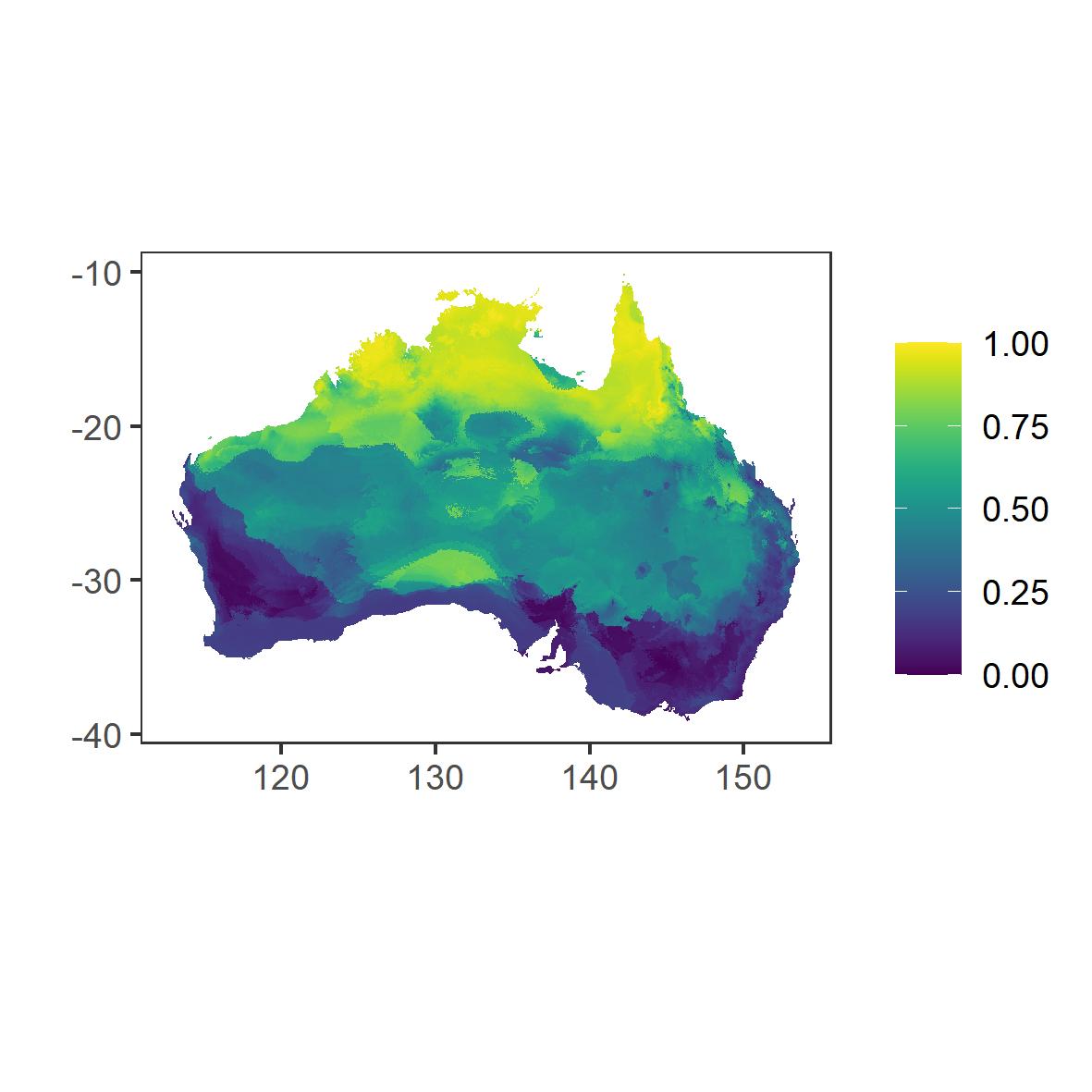


**Supplementary Figure S4** Comparison of climates, using all climatic variables and the multivariate environmental similarity surface (MESS) methods. Positive values indicate cells with similar values to the predictor set, whereas negative values indicate novel climates. Maps were created through R statistical software [78]. The figures were generated in R (version 3.6.0, https://www.r-project.org/).


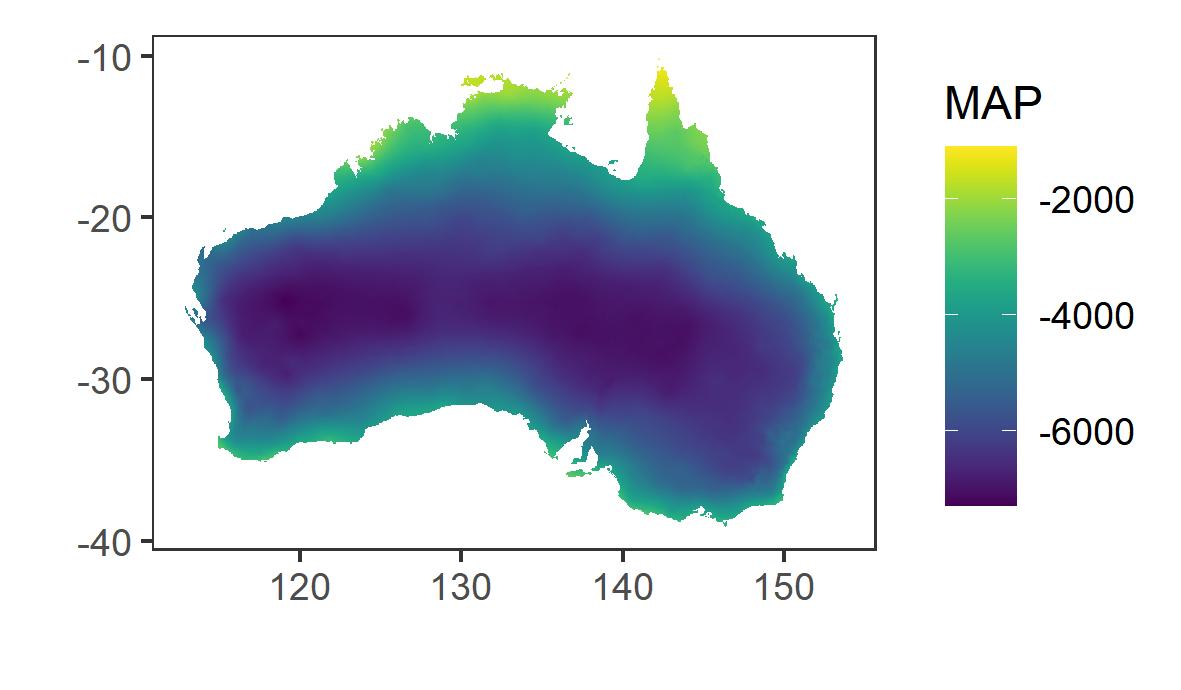

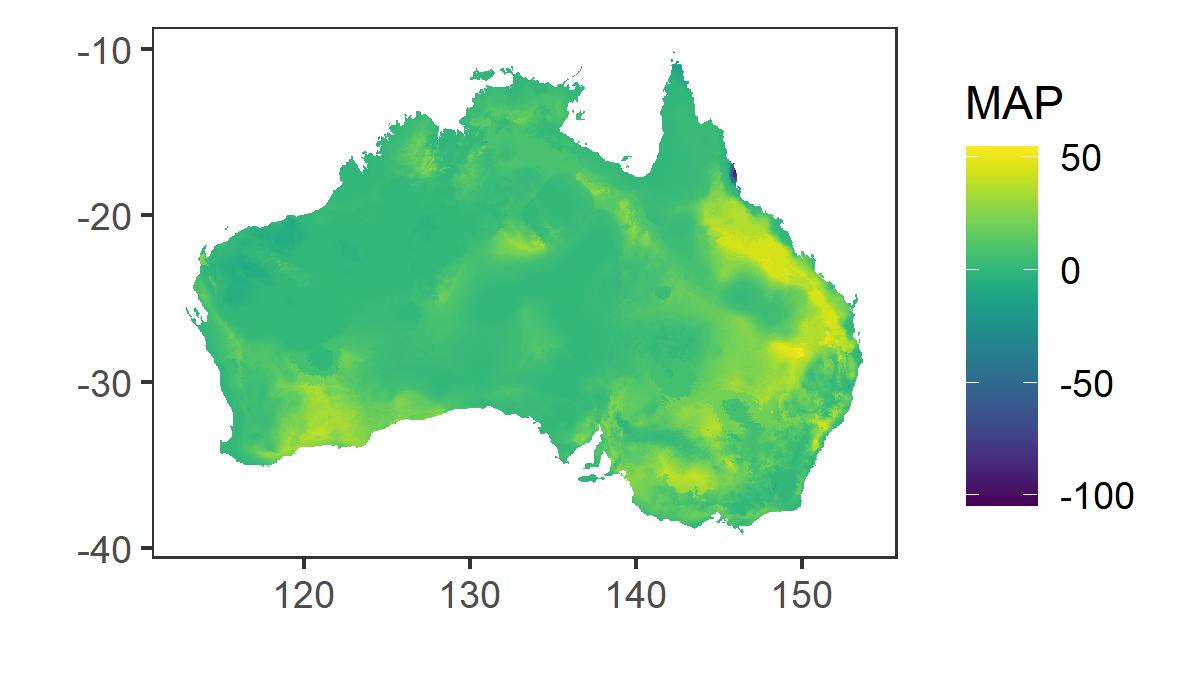

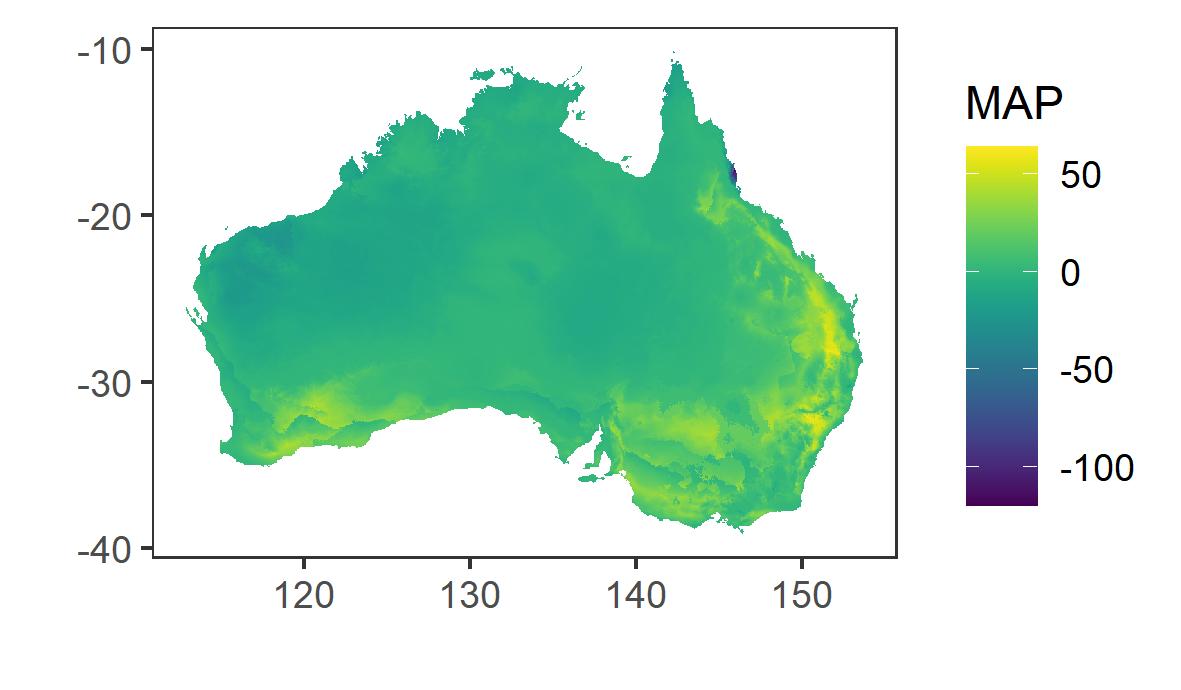

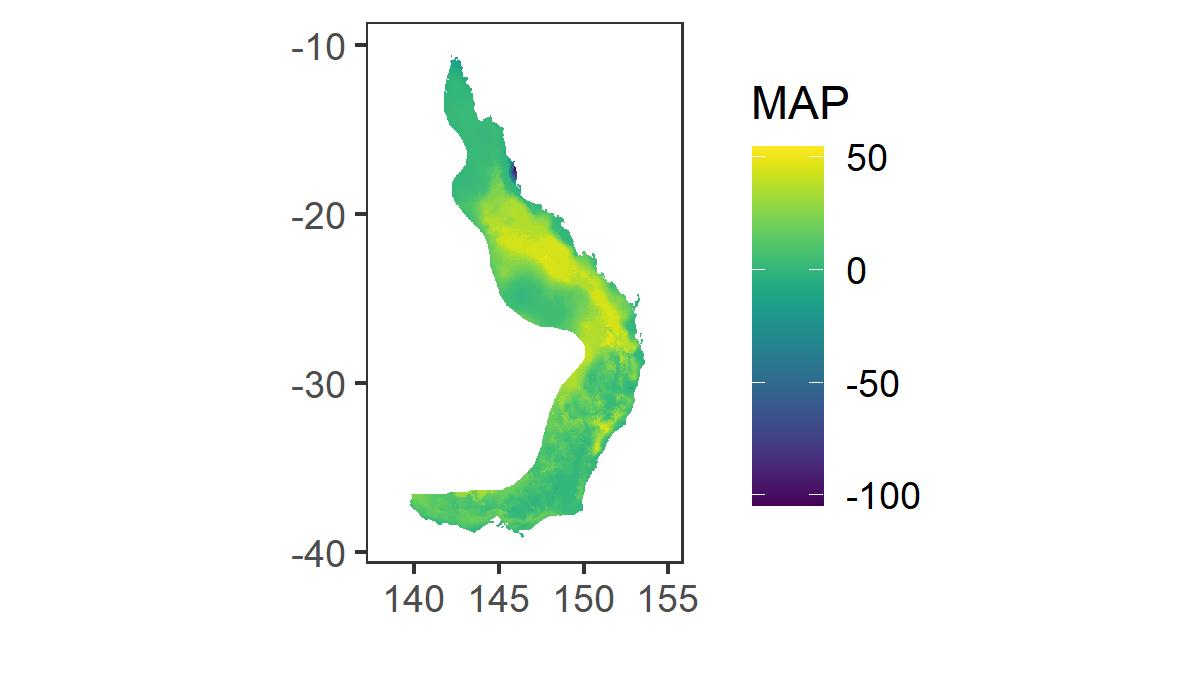

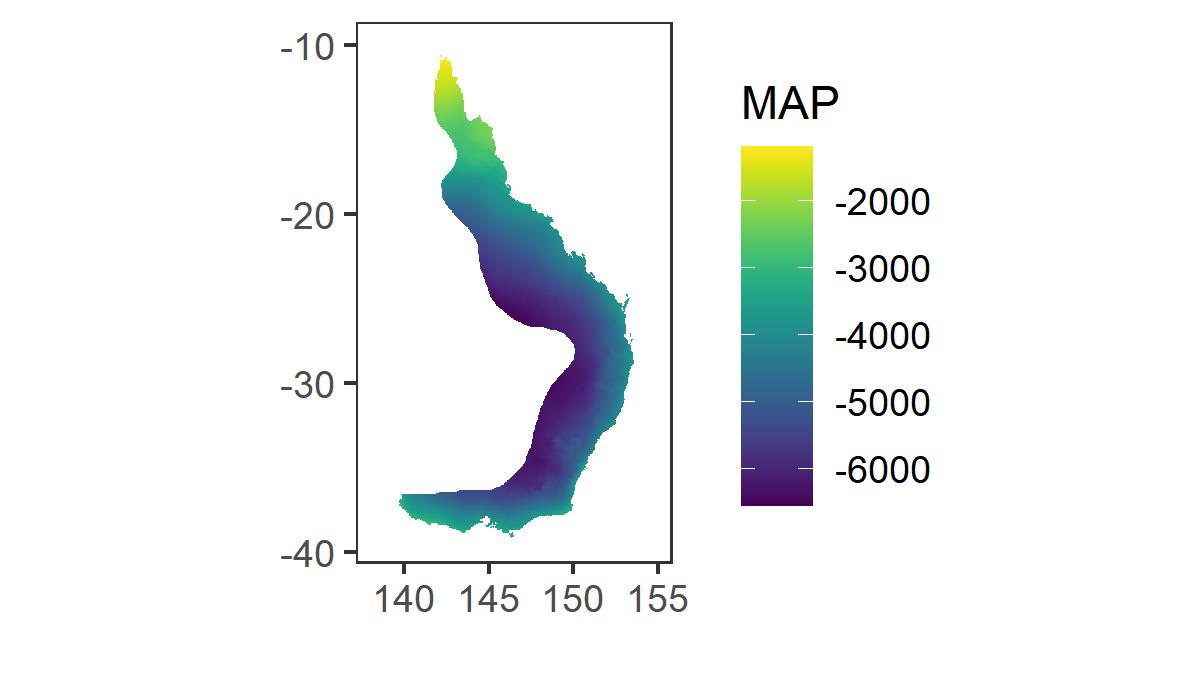

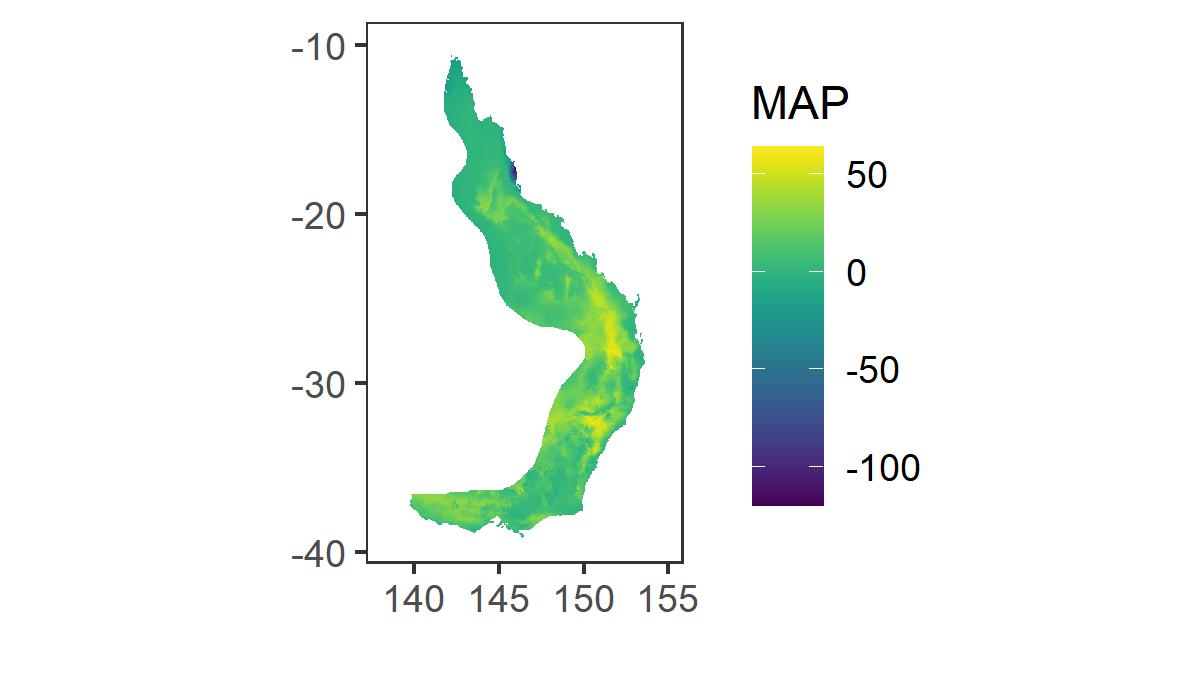


*Great Dividing Range*

*Australia wide*

*Past*

*Current*

*Future*

**Supplementary Figure S5** Analogous climates between current and past climate for selected variables, demonstrated using multivariate environmental similarity surface (MESS) methods. Positive values indicate cells with similar values to the predictor set, whereas negative values indicate novel climates**.** Maps were created through R statistical software [78]. The figures were generated in R (version 3.6.0, https://www.r-project.org/).


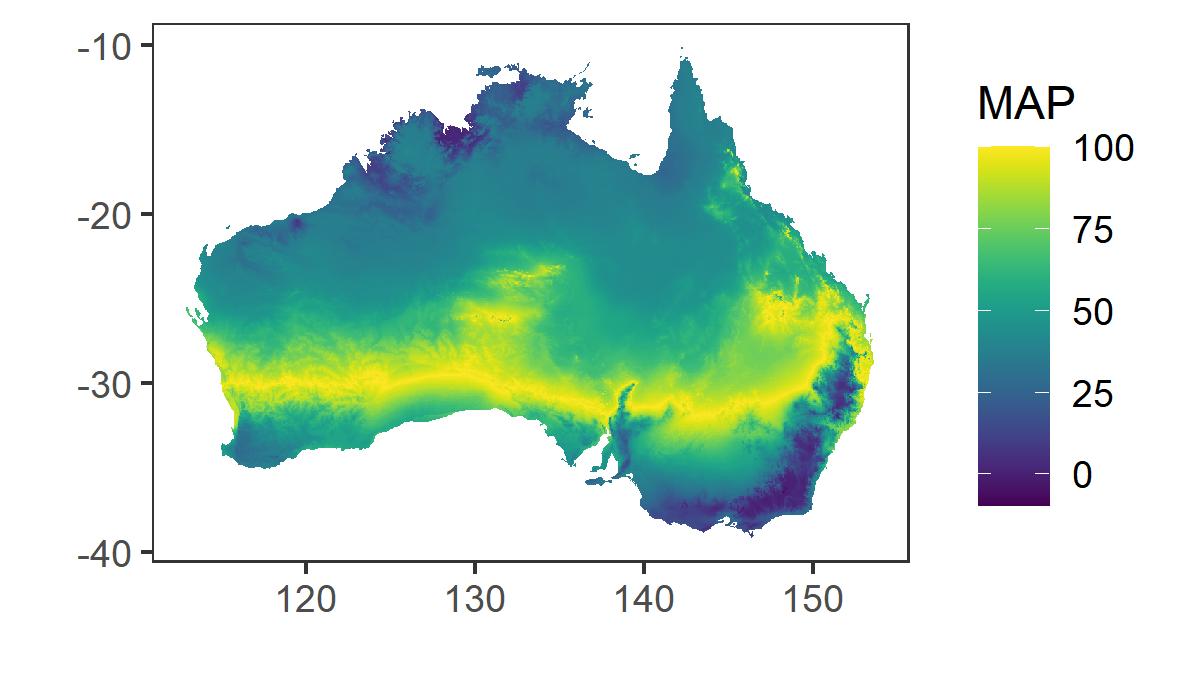

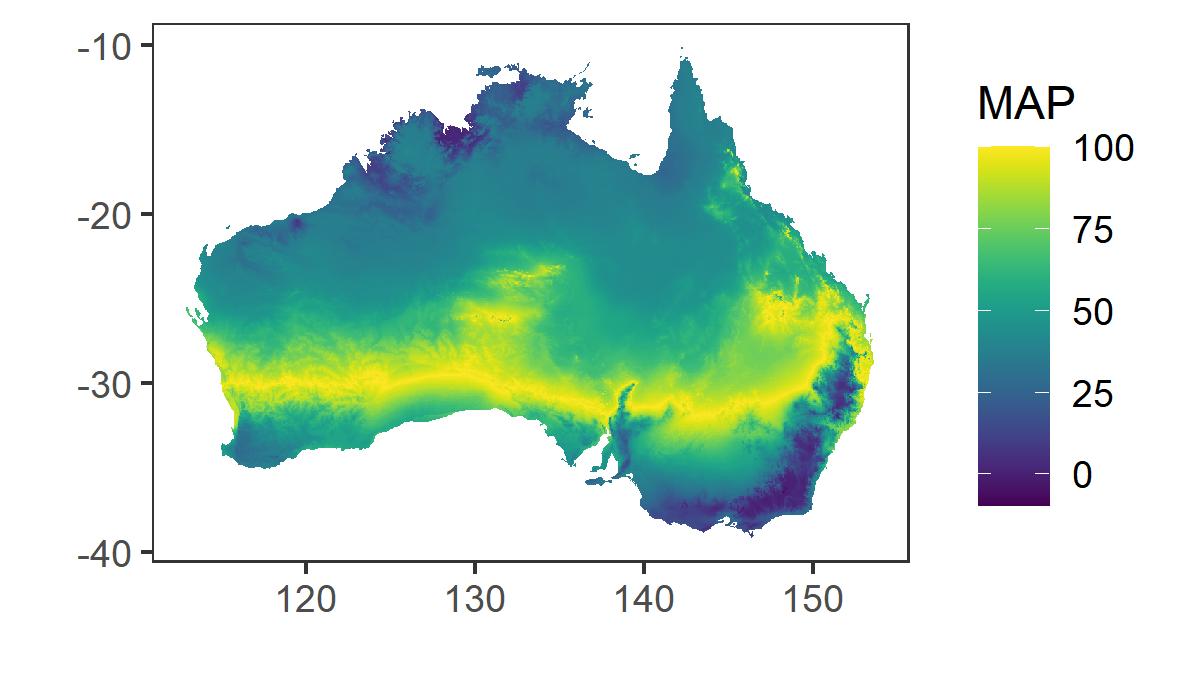

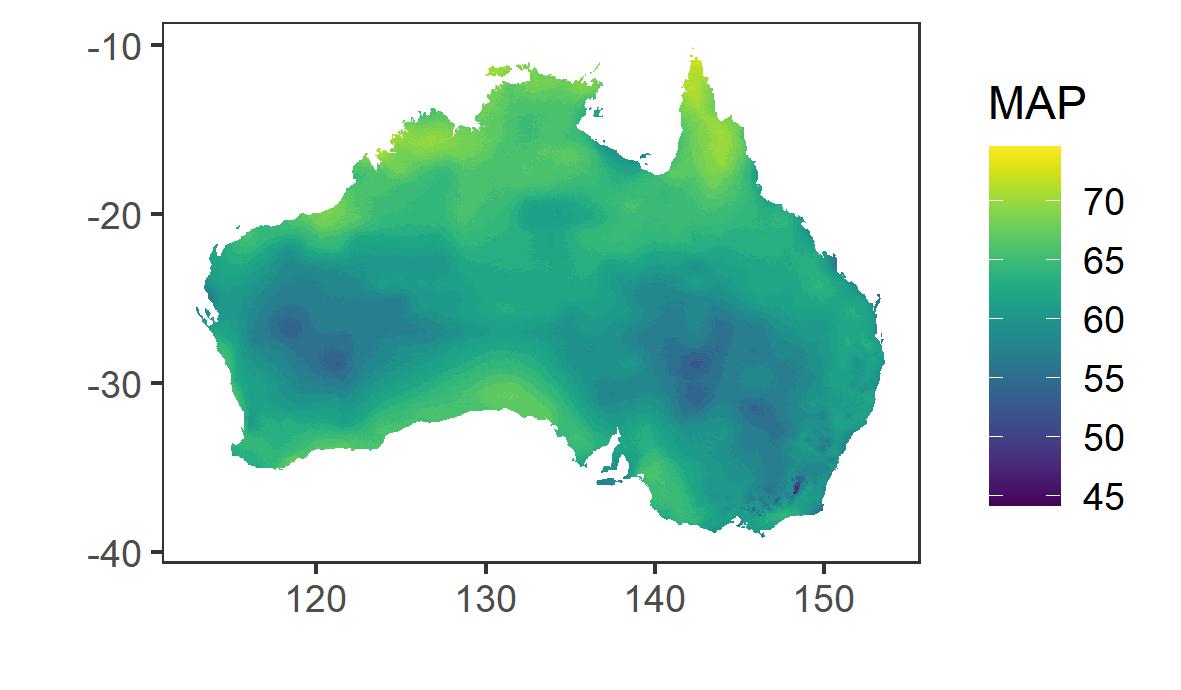

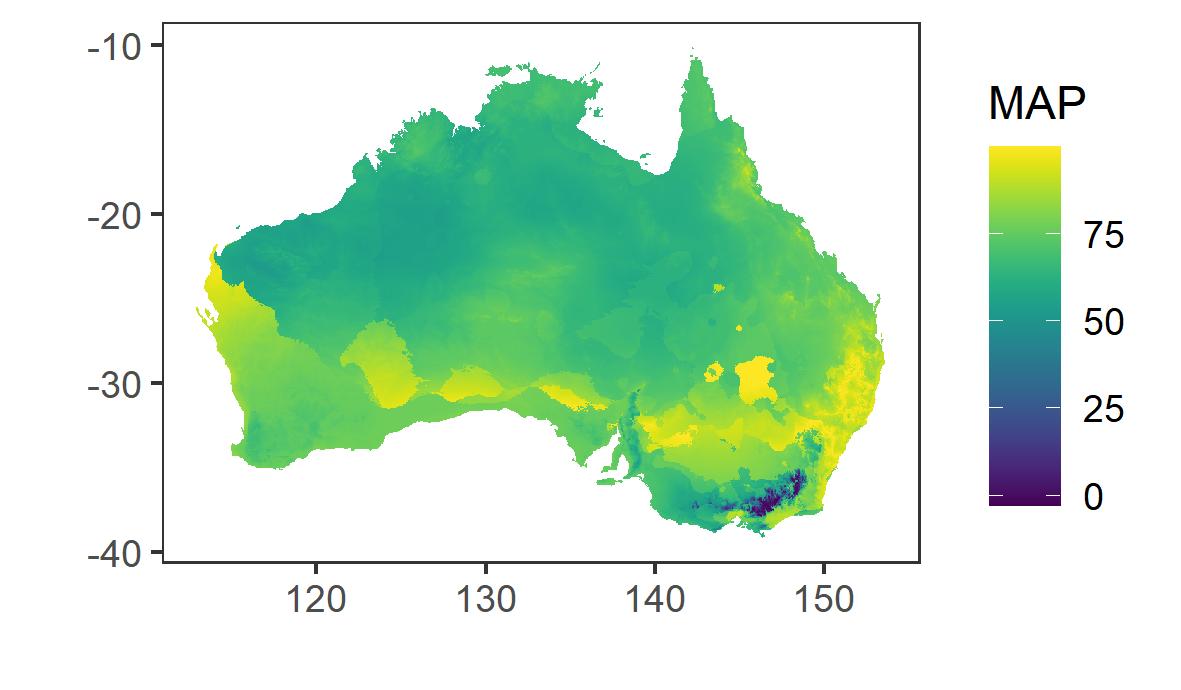

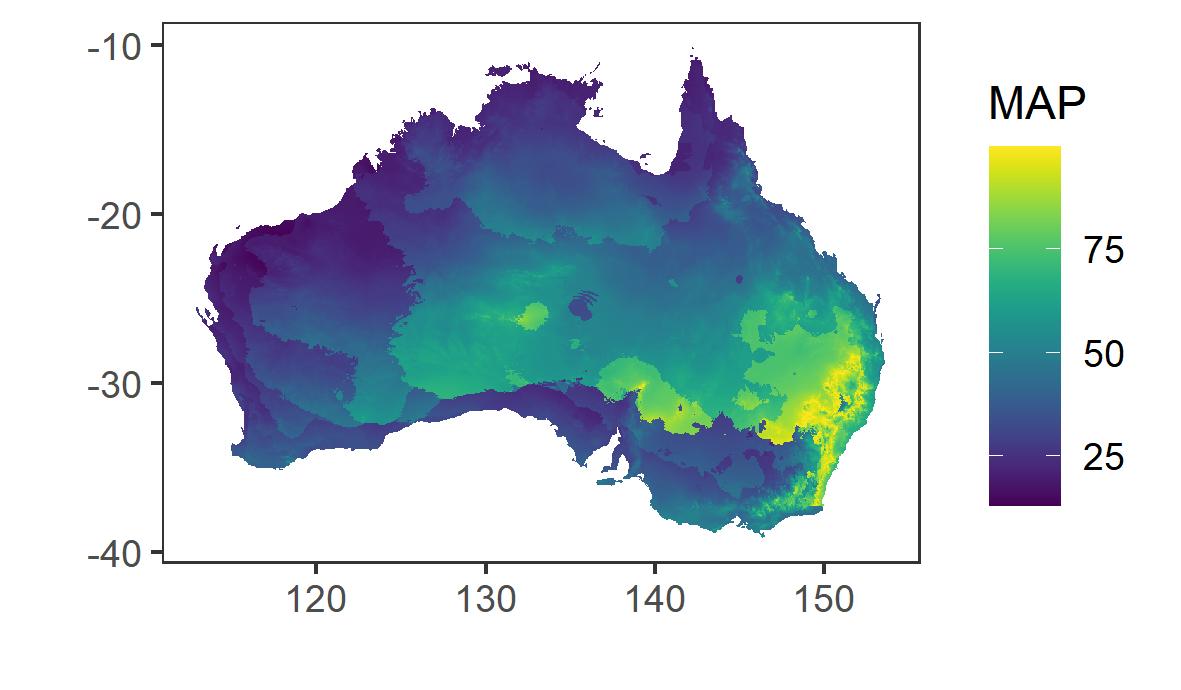


*BIO1 Annual mean temperature*

*BIO13 Precipitation of wettest month*

*BIO19 Precipitation of coldest quarter*

*BIO18 Precipitation of warmest quarter*

**Supplementary Figure S6**. Pairwise correlations (Pearson’s R) for past, present and future climates. Significantly correlated variables are indicate with a cross. The figures were generated in R (version 3.6.0, https://www.r-project.org/).


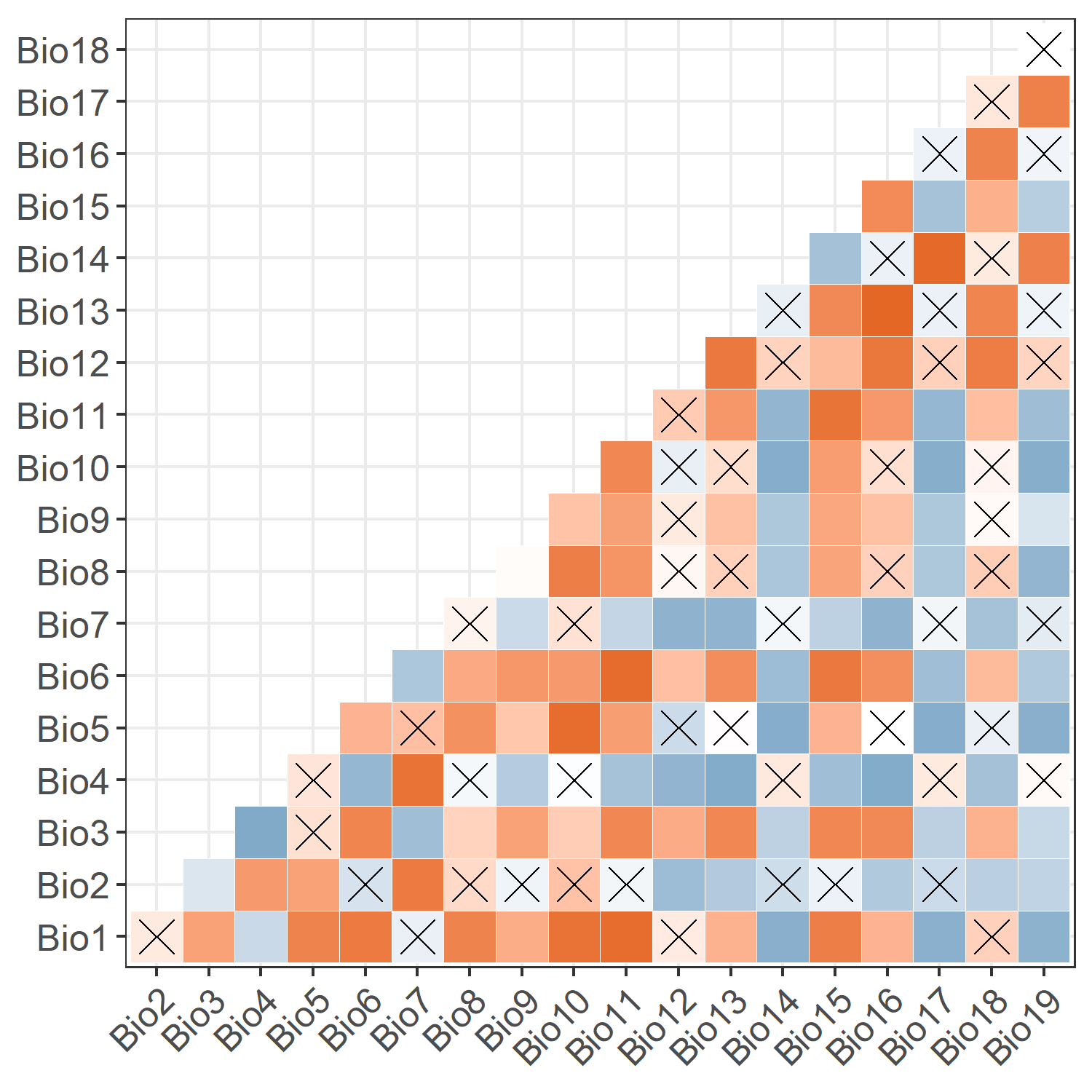


*Future Climate*


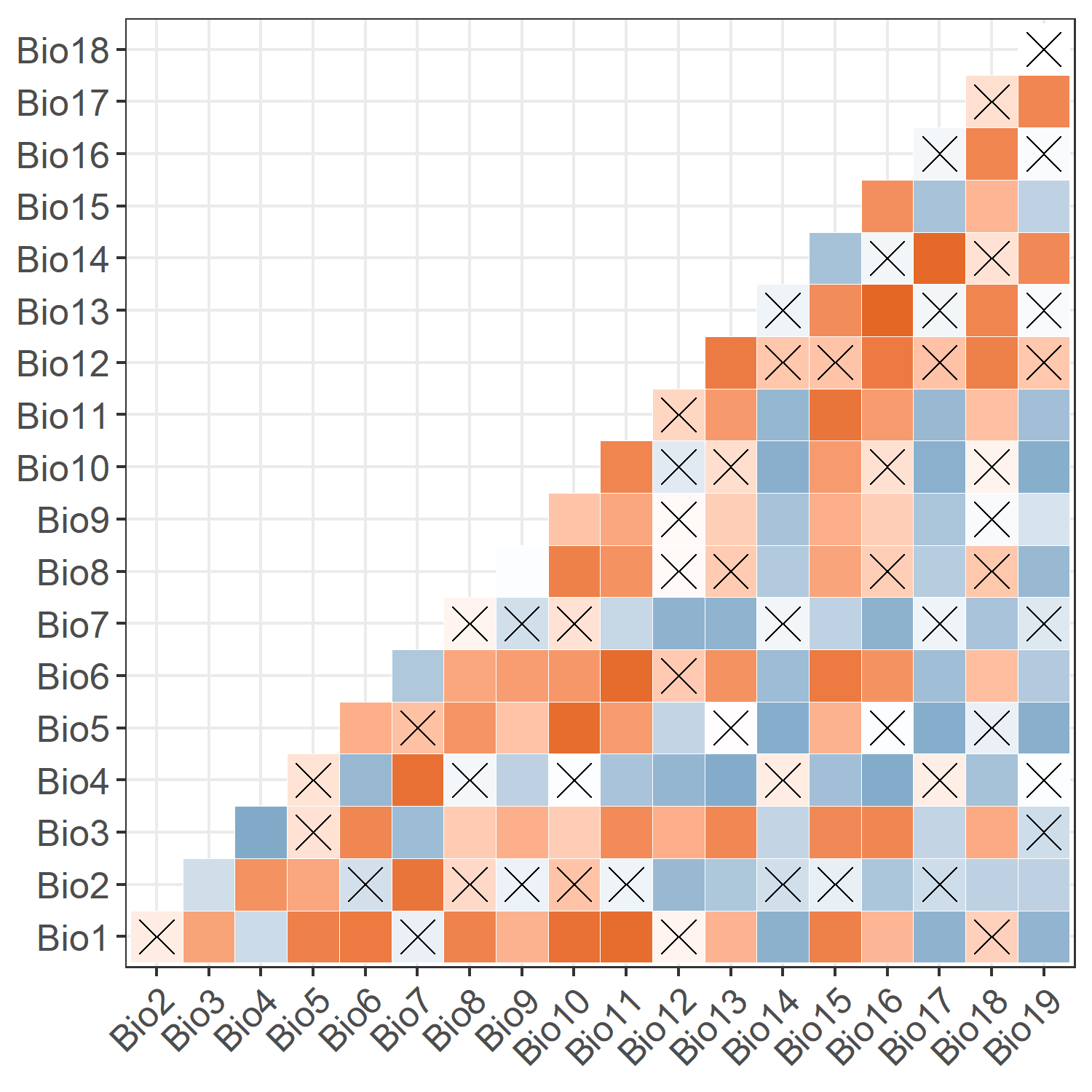


*Current Climate*


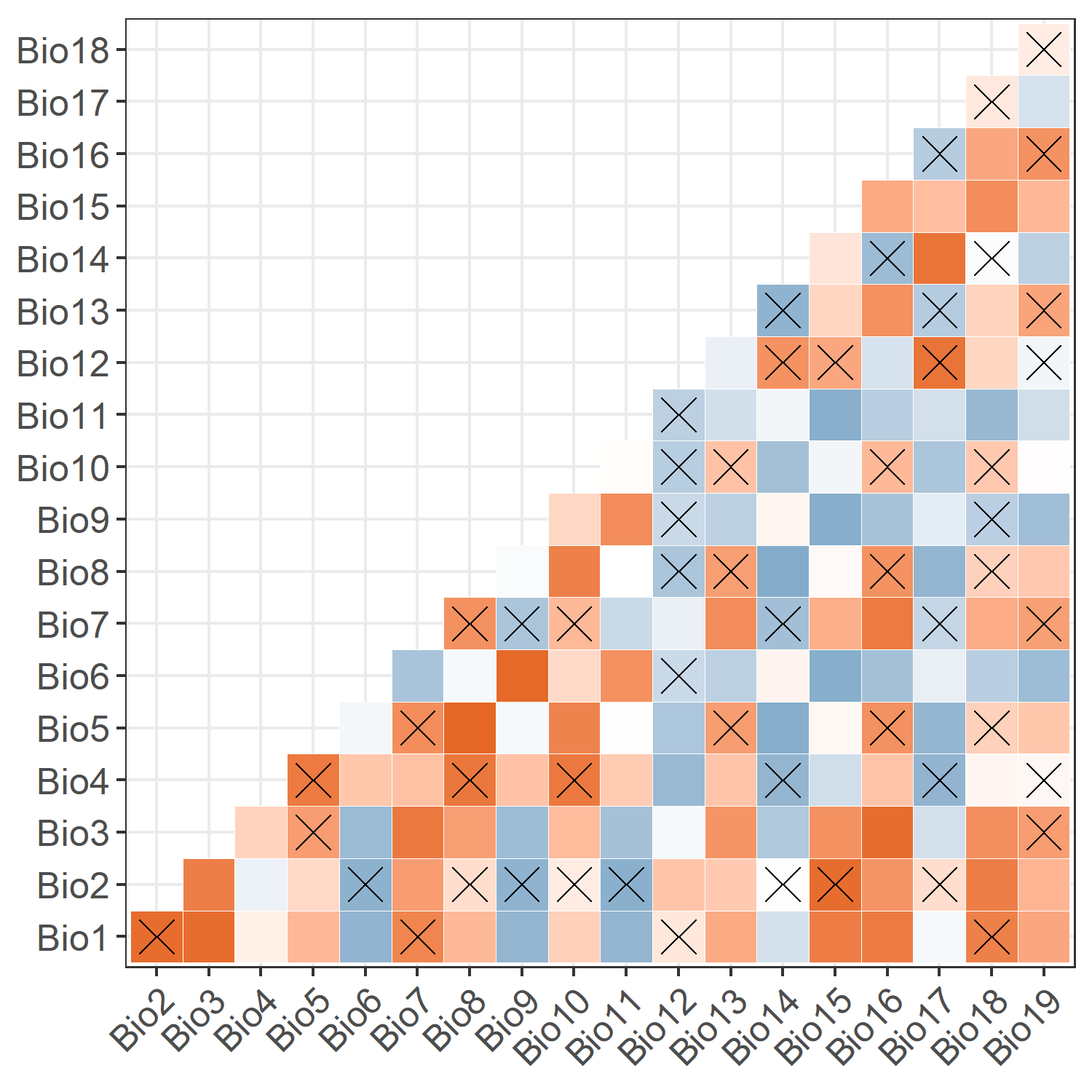


*Past Climate*


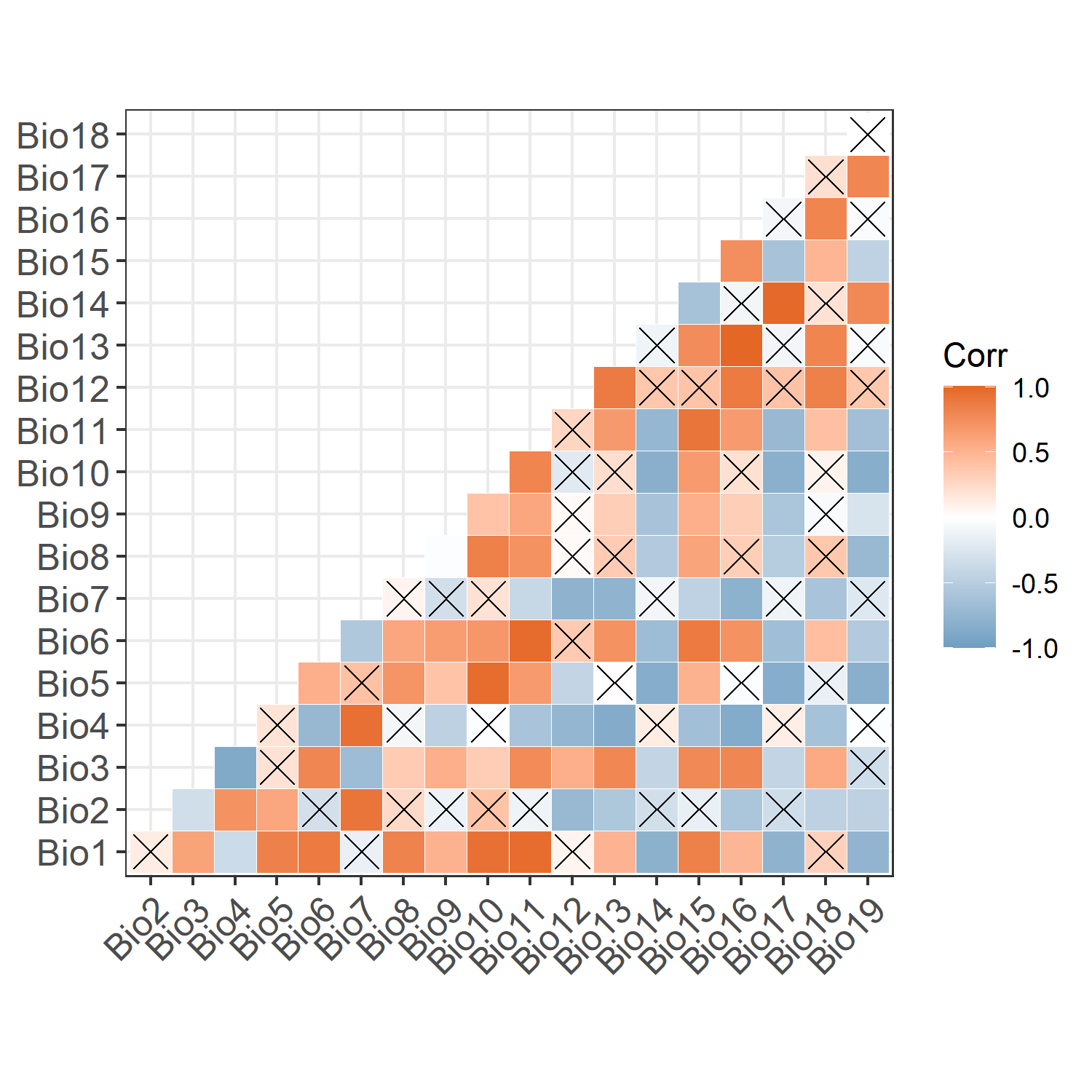

Supplement: Supplementary file 1 — Supplementary Information [file 41598_2020_79551_MOESM1_ESM.docx]
